# Protein co-expression networks identified from HOT lesions of ER+HER2–Ki-67high luminal breast carcinomas

**DOI:** 10.1038/s41598-021-81509-9

**Published:** 2021-01-18

**Authors:** Kimito Yamada, Toshihide Nishimura, Midori Wakiya, Eiichi Satoh, Tetsuya Fukuda, Keigo Amaya, Yasuhiko Bando, Hiroshi Hirano, Takashi Ishikawa

**Affiliations:** 1 Department of Breast Surgery, Tokyo Medical University Hachioji Medical Centre, Tokyo, 193-0998 Japan; 2 Department of Breast Surgery, Tokyo Medical University Hospital, Tokyo, 160-0023 Japan; 3 Department of Translational Medicine Informatics, St. Marianna University School of Medicine, Kanagawa, 216-8511 Japan; 4 Department of Diagnostic Pathology, Tokyo Medical University Hachioji Medical Centre, Tokyo, 193-0998 Japan; 5 Department of Pathology, Institute of Medical Science, Tokyo Medical University, Tokyo, 160-0023 Japan; 6 Research and Development, Biosys Technologies Inc, Tokyo, 152-0031 Japan

**Keywords:** Cancer, Cell biology, Computational biology and bioinformatics, Molecular biology, Diseases, Medical research, Oncology

## Abstract

Patients with estrogen receptor-positive/human epidermal growth factor receptor 2-negative/Ki-67-high (ER+HER2–Ki-67high) luminal breast cancer have a worse prognosis and do not respond to hormonal treatment and chemotherapy. This study sought to identify disease-related protein networks significantly associated with this subtype, by assessing in-depth proteomes of 10 lesions of high and low Ki-67 values (HOT, five; COLD, five) microdissected from the five tumors. Weighted correlation network analysis screened by over-representative analysis identified the five modules significantly associated with the HOT lesions. Pathway enrichment analysis, together with causal network analysis, revealed pathways of ribosome-associated quality controls, heat shock response by oxidative stress and hypoxia, angiogenesis, and oxidative phosphorylation. A semi-quantitative correlation of key-protein expressions, protein co-regulation analysis, and multivariate correlation analysis suggested co-regulations via network-network interaction among the four HOT-characteristic modules. Predicted highly activated master and upstream regulators were most characteristic to ER-positive breast cancer and associated with oncogenic transformation, as well as resistance to chemotherapy and endocrine therapy. Interestingly, inhibited intervention causal networks of numerous chemical inhibitors were predicted within the top 10 lists for the WM2 and WM5 modules, suggesting involvement of potential therapeutic targets in those data-driven networks. Our findings may help develop therapeutic strategies to benefit patients.

## Introduction

Breast cancer is heterogeneous and comprises diverse biological subtypes that respond differently to primary therapies and clinical outcomes^1^. The 12th St. Gallen International Breast Cancer Conference (2011) Expert Panel adopted the classification of five molecular subtypes of invasive breast cancer which have been differentiated using the immunohistochemistry (IHC)-based expression of estrogen receptor (ER), progesterone receptor (PgR), human epidermal growth factor receptor 2 (HER2), and Ki-67^2^. Ki-67 is a nuclear marker of cell proliferation, and its high expression levels in breast cancer are associated with worse outcomes. Currently, Ki-67 measurement is not included in routine clinical decision-making due to a lack of clarity regarding its exact role. Recent studies indicated that a decrease in Ki-67 expression after neoadjuvant endocrine treatment may predict long-term outcome^3^. There is also an urgent need to standardize the analysis of Ki-67 expression and validate its clinical utility. Notably, tumor heterogeneity remains a potential issue. The luminal-B subtype is defined by IHC as ER-positive/HER2-negative/Ki-67-high (ER+HER2–Ki-67high) tumors. However, this definition does not include all luminal-B tumors, among which approximately 6% are negative for both ER and HER2 (ER−HER2−). Moreover, the Ki-67 cut-off point to distinguish luminal A and luminal B has not been standardized^1^.

Luminal-B tumors comprise 15–20% of breast cancers, and are characterized by a more aggressive phenotype, higher histological grade and proliferative index, and a worse prognosis. This subtype is associated with a higher recurrence rate and lower survival rates after relapse compared with the luminal-A subtype. The Ki-67 labelling index is a clinically validated prognostic factor in early breast cancer. In the neoadjuvant setting, it predicts the likelihood of pathological complete response to chemotherapy. Furthermore, Ki-67 in the residual tumor and changes in the Ki-67 labelling index between primary and residual tumors are prognostic factors for long-term outcomes^4^. At the Tokyo Medical University Hospital Hachioji Medical Centre (Hachioji, Tokyo, Japan) 671 patients with breast cancer received surgical operation between 2015 and 2019. Among these cases, the proportions of subtypes were as follows: luminal-A (58%), luminal-B (20%), luminal-HER2 (11%), HER2 (2%), and triple-negative (9%) (Fig. S1a). The luminal-B cases showed a worse recurrence-free survival compared with the luminal-A cases (log-rank test: *p* < 0.0000001) (Fig. S1b). The ER + HER2 − luminal B cases with high Ki-67 (> 50%) were found to be the high-risk group (log-rank test: *p* < 0.005) among luminal B cases (Fig. S1c). Of note, high Ki-67 (> 70%) was measured in only 21 of those cases (16%).

Advances in high accuracy mass spectrometry (MS) has rendered clinical proteomics feasible to perform shotgun sequencing and quantitative analysis of proteins expressed in clinical specimens. Proteome data obtained through this approach can be used to identify key disease-related proteins and therapeutic targets^5^. A laser microdissection (LMD) technique enables the collection of target cells of a certain type from sections of formalin-fixed paraffin-embedded (FFPE) cancer tissue (Fig. S2)^6, 7^. Label-free spectral counting and identification-based semi-quantitative shotgun proteomic analysis of microdissected target cancerous cells of a certain type, that characterized luminal B breast cancer tumors of ER+HER2− and Ki-67 score (> 80%), were used. Weighted correlation network analysis, termed weighted gene co-expression network analysis (WGCNA)^8^, is an extensively applied unsupervised clustering method based on the correlation network of gene and/or protein expression^9–13^. This study aimed to identify disease-related protein networks associated with HOT lesions, for which the WGCNA pipeline and hypergeometric-based over-representative analysis (ORA) was applied.

## Results

### Proteome datasets of ER+HER2–Ki-67high luminal breast carcinomas

MS-based proteomic analysis was performed using 10 microdissected lesions comprising five HOT and five COLD spots obtained from the five FFPE tissue specimens of this subtype. These specimens were selected for their preserved condition, tumor area, and well-clarified pathological diagnosis and molecular marker status (ER+HER2–Ki-67high (> 80%)) (Table 1). Pre-surgical treatment was not performed in any of the cases.

**Table 1:** Clinicopathological information of patients with luminal B breast cancer.

Patient No	Sample ID (Ki-67 score) A, HOT lesion; B, COLD lesion	Age	Tumor location	Tumor size on CT (mm)	TNM classification			Clinical stage	Biomarkers & status			
					c-T	c-N	c-M		ER, %	PgR, %	HER2	Ki-67, %
Patient 1	1A (69.2%) 1B (20.1%)	78	L) DCE	20 × 20 × 6	T4b	N1	M0	IIIB	+ 50	0	− 0	90
Patient_2	2A (75.6%) 2B (14.3%)	63	L) C	42 × 32 × 21	T2	N1	M0	IIB	+ 80	20	− 1	80
Patient_3	3A (58.9%) 3B (13.0%)	80	L) D	25 × 22 × 15	T2	N1	M0	IIB	+ 100	70	− 1	90
Patient_4	4A (86.4%) 4B (54.0%)	52	L) C	25 × 20 × 15	T2	N0	M0	IIA	+ 90	0	− 1	80
Patient_5	5A (66.7%) 5B (51.3%)	44	L) C'	2*	T1a	N2	M0	IIIA	+ 90	0	− 0	80

*CT* computed tomography; *HER2* human epidermal growth factor receptor 2; *PgR* progesterone receptor; *TNM* tumor-node-metastasis.

*This is a case corresponding to so-called occult cancer. That is, the primary lesion was small and the metastatic lesion was found first. pT1aN2aM0: High malignant type of Grade 3, Ki-67 high in stage IIIA. Lymph node metastases were first discovered. The surgical specimen was n (5/19), but the size of the invasive cancer (infiltration diameter) was only 2 mm and 1 mm. The distance between the two was 3 mm, but lymphatic invasion was remarkable.

A total of 1,862 proteins were significantly identified from all the HOT and COLD lesions; of these, approximately 65% were commonly expressed (Fig. S3a). The proportion of proteins unique to HOT lesions was approximately 26%, whereas that of proteins expressed in the COLD lesions was approximately 10%. Overall, 476 and 180 proteins were characteristic of the HOT and COLD lesions, respectively. Gene ontology (GO) analysis was performed on the GO Biological process, GO Molecular Function, and Protein class using the Protein Analysis THrough Evolutionary Relationships (PANTHER) version 14.1 software program (Paul D. Thomas, University of Southern California, Los Angeles, CA, USA)^14^. Results of the GO analysis were similar between the HOT and COLD groups (Fig. S3b). Proteins were abundantly associated with cellular process, localization, cellular component organization or biogenesis, biological regulation, metabolic process and response to stimulus, in biological process (GO); binding and catalytic activity in molecular function (GO); cytoskeletal protein, transporter, nucleic acid-binding protein, protein-binding activity modulator, translational protein, metabolite interconversion enzyme, protein modifying enzyme, membrane traffic protein, and chaperone in protein class (GO). The fold changes and relative abundances of all identified proteins for both HOT and COLD lesions are visualized in Fig. S4.

### Identification of data-driven key protein networks by WGCNA

**Figure 1:**
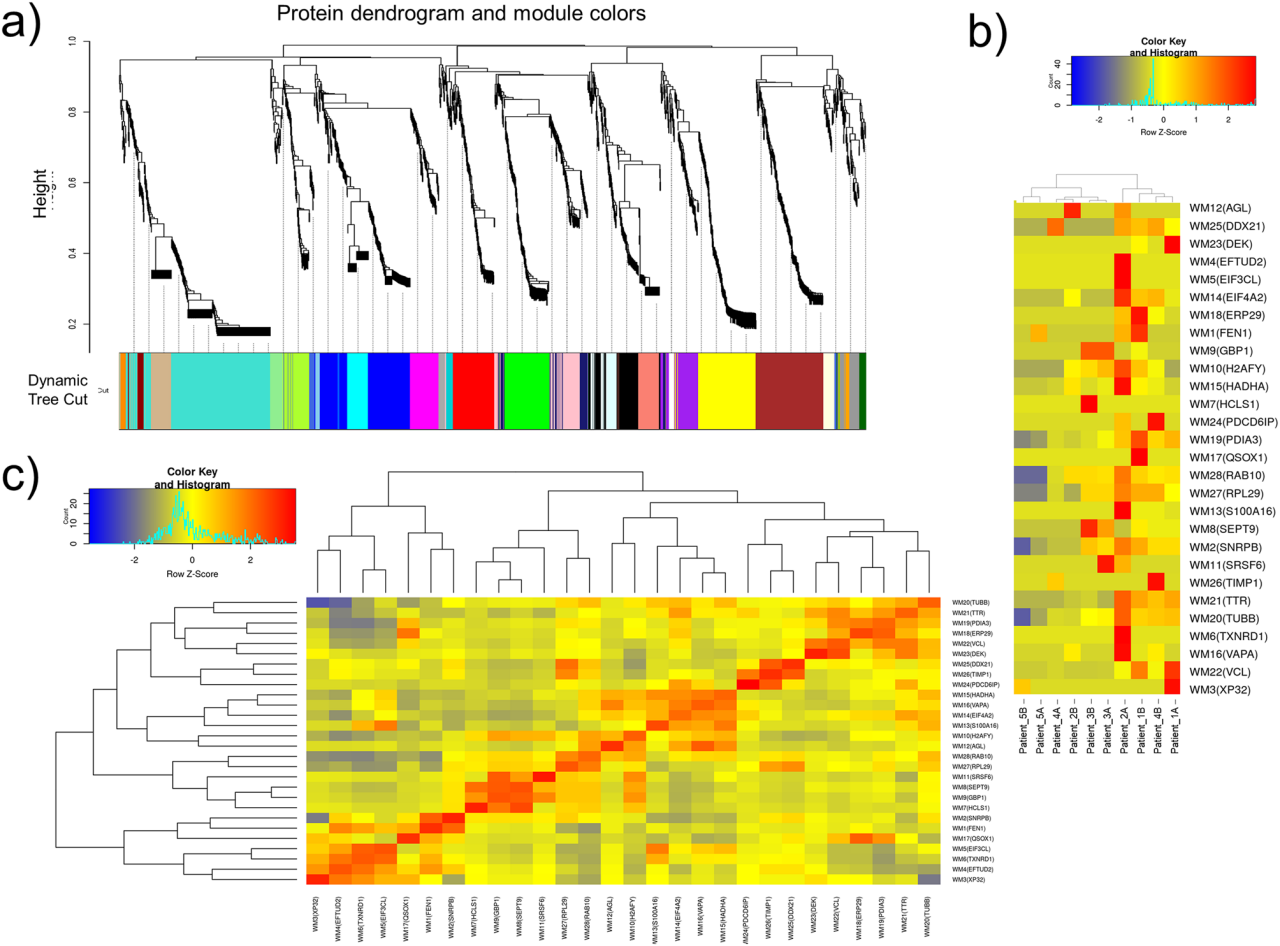
Protein modules identified by weighted gene co-expression network analysis (WGCNA). (**a**) Protein dendrogram obtained by clustering the dissimilarity based on consensus topological overlap with the corresponding module. Colored rows correspond to the 28 modules identified. (**b**) Dendrogram of consensus module eigen-proteins obtained on the consensus correlation. (**c**) Pairwise correlations between the modules in the heatmap of eigen-protein expressions.

Co-expression protein networks are defined as undirected, weighted networks. Modules are clusters of highly interconnected proteins, which correspond to clusters of proteins with high absolute or positive correlations^8^. We identified 28 protein modules by constructing a weighted correlation network and clustered all the identified proteins (Fig. 1). A soft threshold power of 10 was selected to define the adjacency matrix according to the criteria of approximate scale-free topology, with a minimum module size of 5 and a module detection sensitivity *deepSplit* of 4. The correlations between resultant modules and two clinical traits (HOT and COLD) were determined to identify protein modules with upregulated or downregulated expression in the HOT or COLD lesion samples. We could not identify modules which were significantly correlated with clinical traits (i.e., |r|> 0.5 and *p* < 0.05) throughout the 28 modules identified (Fig. S5). However, several WGCNA modules exhibited moderate correlations characteristically with the clinical trait − HOT (|r|> 0.4), which includes WM2 (light green), WM5 (cyan), WM6 (tan), and WM20 (dark grey). The WM5 (cyan) module indicated the highest correlation and significance of its member proteins within the module membership versus gene significance (*Corr*. = 0.96, *p* = 7.4 × 10^−30^) (Fig. S5).

### Statistical ORA

**Figure 2:**
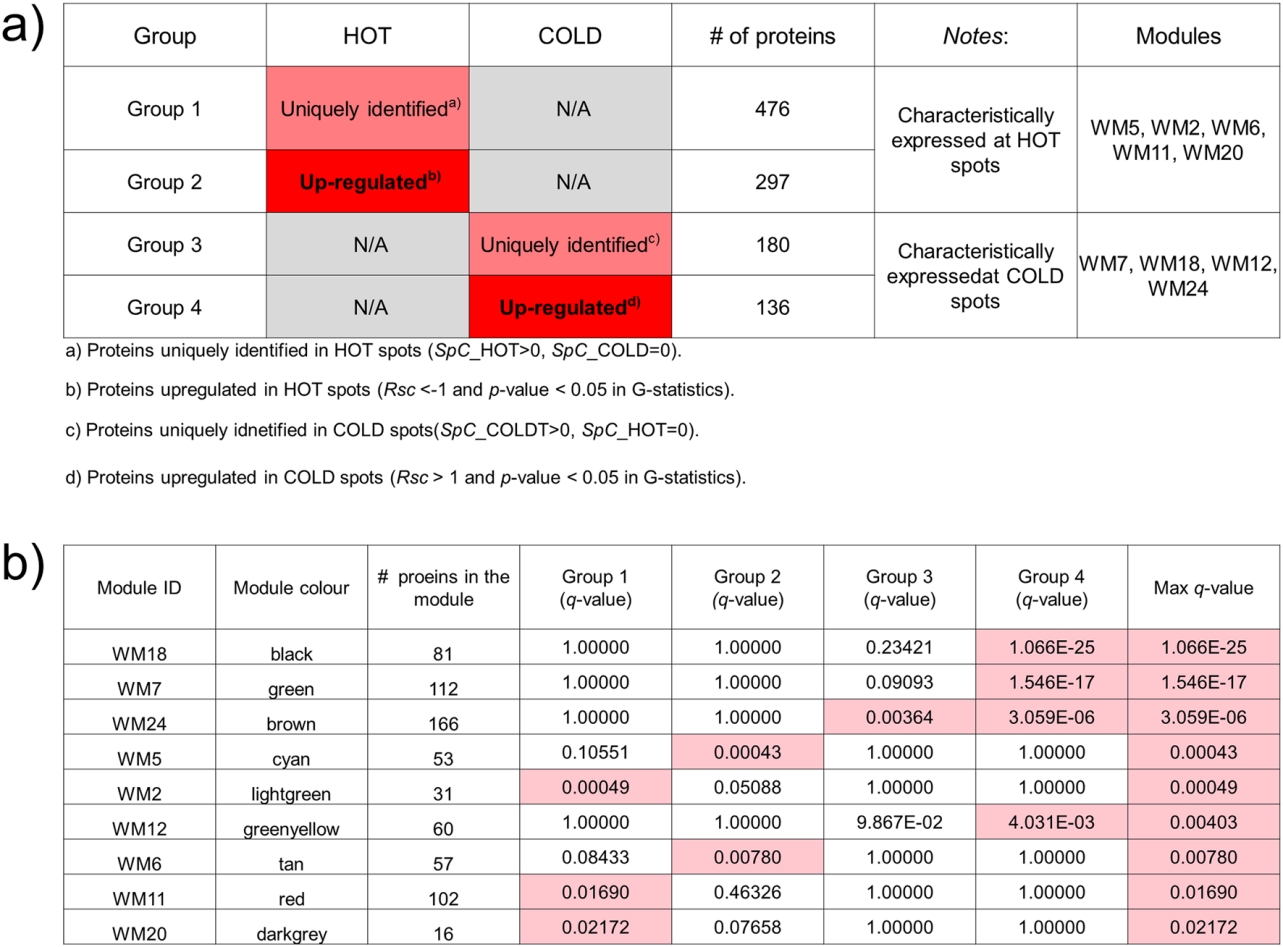
Overlapping proteins unique to the clinical traits and/or upregulated under the HOT or COLD traits, and those from the weighted gene co-expression network analysis (WGCNA). (**a**) Results of identified proteins and spectral counting-based semi-quantitative comparison. Each row represents results for each protein group. The red and pink cells in the HOT and COLD columns indicate that the proteins in the group are uniquely expressed and significantly upregulated, respectively (upregulated with |*Rsc*|> 1 (HOT > COLD or HOT < COLD). The fourth column shows the number of proteins in each protein group. The fifth column provides notes for each protein group. The WGCNA modules with significant overlap with each protein group are listed in the sixth column (‘Modules’ column). (**b**) Overlap in proteins between the groups according to the protein expression profiles and the modules by WGCNA. Each row in the embedded table represents the overlap analysis results for each module. The first and second columns in the table represent module ID and color name of the module, respectively. The third column represents the number of proteins in each module. The fourth, fifth, sixth, and seventh columns indicate the *q* values for overlap in proteins between a module by WGCNA and the four protein groups. In the five columns, significant *q*-values are highlighted in red. The eighth column represents the value of the most significant *q*-value (max *q*-value) in each module. The nine modules with max *q*-values < 0.05 are listed in order.

Trait correlation analysis between eigen components of WGCNA modules and clinical traits often tends to overlook important modules for investigating disease-related molecular networks. Regarding clinical traits which are quite close, such as HOT and COLD in this study, a difficulty in trait-module relationship would be encountered to attain a high significance in the identification of key WGCNA modules. Statistical ORA^13^ would assist in evaluating potential key WGCNA modules with identified proteins uniquely expressed and upregulated in the HOT trait. Notably, 476 and 180 proteins were uniquely expressed in the HOT and COLD lesions, respectively; while 297 and 136 proteins were differentially upregulated, respectively, with |*R*_*SC*_|> 1 (higher than twice fold change) and *p* < 0.05 in G-statistics (Figs. S3a and S4). The overlaps between the WGCNA-derived protein modules and identification-based significantly expressed proteins were subsequently assessed using the over-representation test. We confirmed five important WGCNA modules that showed significant overlap (maximum *q*-value among the groups < 0.05) with protein groups uniquely and/or highly upregulated in the HOT trait (Fig. 2a).

### Functional enrichment analysis of WGCNA modules screened by ORA

**Figure 3:**
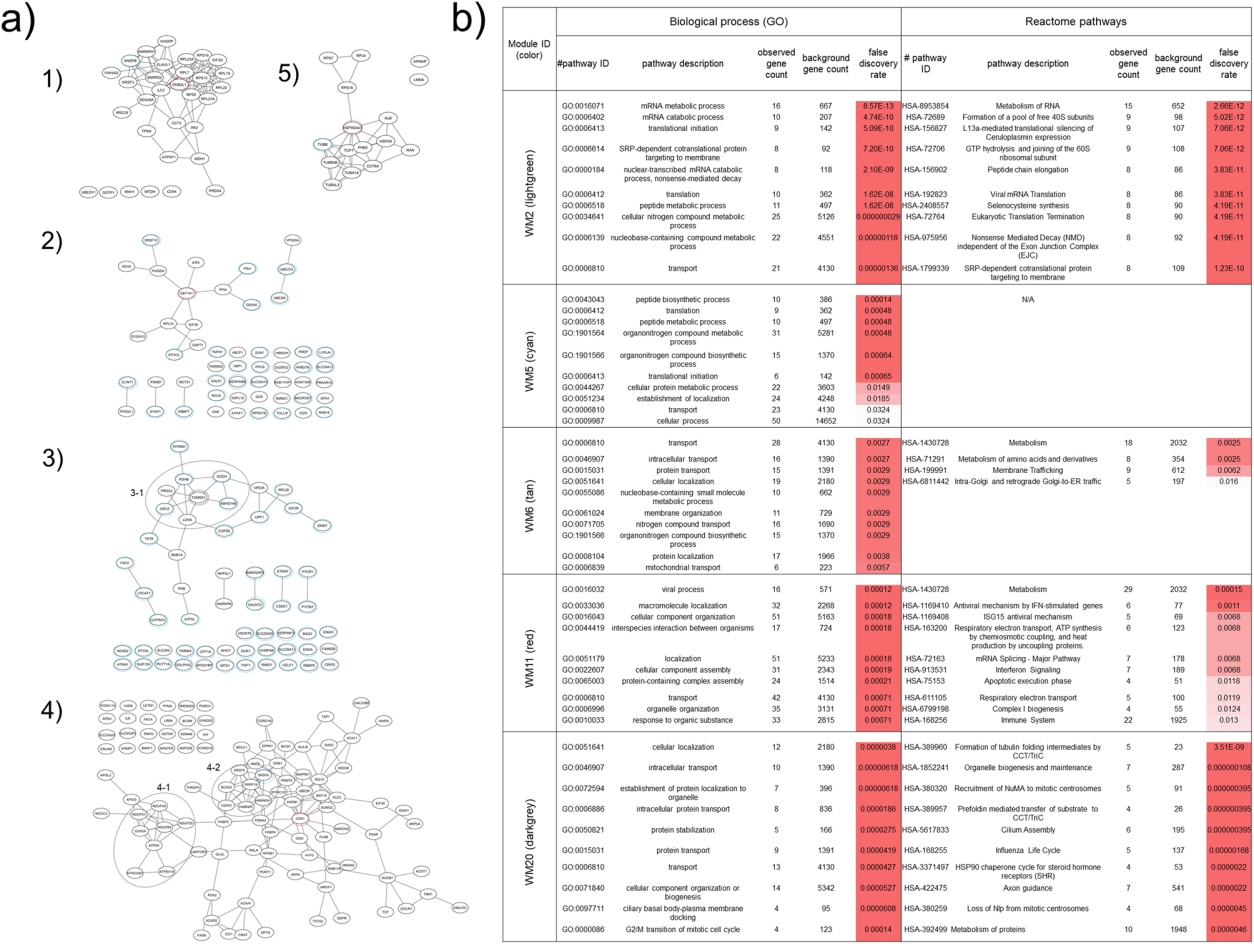
Data-driven protein co-expression networks and pathway enrichment results obtained for the HOT trait. (**a**) The co-expression networks of respective modules: (1) WM2, (2) WM5, (3) WM6, (4) WM11, and (5) WM20 modules. Dotted circle nodes in blue and red represent eigen-proteins and/or hub proteins for the respective module, respectively. (**b**) Top 10 pathways enriched for the protein core networks obtained for biological process (GO) and Reactome pathways.

Our WGCNA analysis followed by ORA-based screening identified the five protein modules significant to proteins expressed uniquely and upregulated in the HOT lesions: WM2 (light green), WM5 (cyan), WM6 (tan), WM11 (red), and WM20 (dark grey) (Fig. 2b). The biological connectivity among the proteins in each module was analyzed by mapping the module proteins in the human protein–protein interaction (PPI) network, and among the biological pathways by pathway enrichment analysis^15^. Figure 3 shows data-driven protein co-expression networks; hub proteins for respective modules identified using the *cytoHubba plugin*^16^ and eigen-proteins are indicated in red and blue dashed circles, respectively, and pathway enrichment results identified for the HOT trait are shown. Similarly, data-driven protein co-expression networks and pathways enriched for the COLD trait are presented in Fig. S6.

The enriched pathways of the WM2 (light green) module included mRNA metabolic process, signal-recognition particle-dependent co-translational protein targeting to membrane, nuclear-transcribed mRNA catabolic process, nonsense-mediated decay, ribonucleoprotein complex, and ribosome (Fig. 3b). The hub protein guanine nucleotide-binding protein subunit beta-2-like 1 (GNB2L1) is known as the receptor of activated protein C kinase 1 (RACK1), which is a component of the 40S ribosomal subunit involved in translational repression and initiation of the ribosome quality control^17^. The module member protein ELAVL1, the RNA-binding protein HuR, is also involved in adenylate-uridylate-rich element-mediated mRNA decay. A breast cancer study using IHC of 160 primary breast carcinomas revealed that higher RACK1 expression was correlated with shorter overall survival, and suggested a correlation between RACK1 and the commonly used clinicopathological biomarkers (e.g., Ki-67 and ER) in breast cancer^18^.

The enriched pathways of the WM5 (cyan) module involved peptide biosynthetic process, translation, organonitrogen compound metabolic process, and translational initiation (Fig. 3b). The hub protein elongation factor 1-alpha 1 (eEF1α1) encoded by *EEF1A1* belongs to the translation factor-related (TRAFAC) class translation factor GTPase superfamily. It strongly promotes the heat shock response, which protects cancer cells from proteotoxic stress, such as oxidative stress and hypoxia^19^. EEF1A1 mRNA levels are downregulated in most breast cancers, and this low expression has been associated with poor prognosis for patients with ER-positive breast cancer^20^. In contrast, the protein eEF1α1 was overexpressed in both ER and progesterone receptor-positive and lymph node-negative ductal breast carcinomas, where it should be noted that mRNA expression and protein expression of EEF1A1 are opposite^20^.

The enriched pathways of WM6 (tan) included intracellular transport, organonitrogen compound biosynthetic process, membrane organization, and metabolism of amino acids and derivatives (Fig. 3b). The subnetwork 3–1 (Fig. 3a) is involved in the oxidation–reduction process, detoxification of reactive oxygen species (ROS), and tumor protein p53 (TP53) regulates metabolic genes, in which thioredoxin reductase 1 (TXNRD1) and peroxiredoxin 2 (PRDX2) are the main members in the network cluster of redox-active center and selenocysteine. TXNRD1, the top hub protein in this module, protects cancerous cells against oxidative stress by reducing thioredoxin 1 (TXN), which reduces oxidized cysteines in cellular proteins and scavenges peroxides by PRDX^21^. TXNRD1 is upregulated in many tumors, such as oral squamous cell carcinoma^22^, lung cancer^23^, and breast cancer^24^. The enzymatic TXN/TXNRD1 system involves a key mechanism governing S-nitrosothiol homeostasis. However, the ER-positive status of breast tumors has been associated with significantly lower levels of TXNDR1 protein expression^24^. Indeed, we also observed the low expression of TXNDR1 compared with TXN in this study, which may be relevant to the HER2-negative breast carcinomas. High TXN expression enhances angiogenesis through upregulation of protein expression of hypoxia-inducible factor 1alpha (HIF-1α) and vascular endothelial growth factor (VEGF). It also inhibits apoptosis by binding to the pro-apoptotic proteins, apoptosis signal-regulating kinase 1 (ASK1) and phosphatase and tensin homolog (PTEN), which promote cancer cell growth^21^. It was suggested that an impairment of the TXN/TXNRD1enzymatic system is associated with the development of resistance against hormonal therapies.

The enriched pathways of the WM11 (red) module included macromolecule localization, interspecies interaction between organisms, respiratory electron transport, adenosine triphosphate synthesis by chemiosmotic coupling, and heat production by uncoupling proteins, and mRNA Splicing—Major Pathway (Fig. 3b). The subnetworks 4–1 and 4–2 are involved in oxidative phosphorylation and mRNA Splicing—Major Pathway, respectively (Fig. 3a). The hub protein cyclin-dependent kinase 1 (CDK1) plays a key role in regulating cell cycle progression, and is a potent therapeutic target for inhibitors in the treatment of cancer. MYC has been an attractive therapeutic target for the treatment of breast cancer, whereas a direct inhibition of MYC remains challenging. Kang et al. recently proposed that targeting of CDK1, but not CDK4/6 or CDK2, could be selectively lethal to MYC-dependent human breast cancer cells^25^. CDK1 is currently considered the best CDK target for breast cancer therapy^26^.

The WM20 (dark grey) module was enriched with pathways including the establishment of protein localization to organelle, protein targeting, and extracellular exosome (Fig. 3b). The top hub proteins in this module included heat shock protein (HSP) HSP90-alpha (HSP90AA1), T-complex protein 1 subunit alpha (TCP1/CCT1), and T-complex protein 1 subunit zeta (CCT6A), which also belong to HSPs. HSPs play a crucial role as molecular chaperones under carcinogenic stress conditions. Overexpression of HSP90AA1, TCP1/CCT1, and CCT6A results in poor survival for patients with breast cancer^27, 28^.

### Semi-quantitative protein expression and multivariate correlation analysis

**Figure 4:**
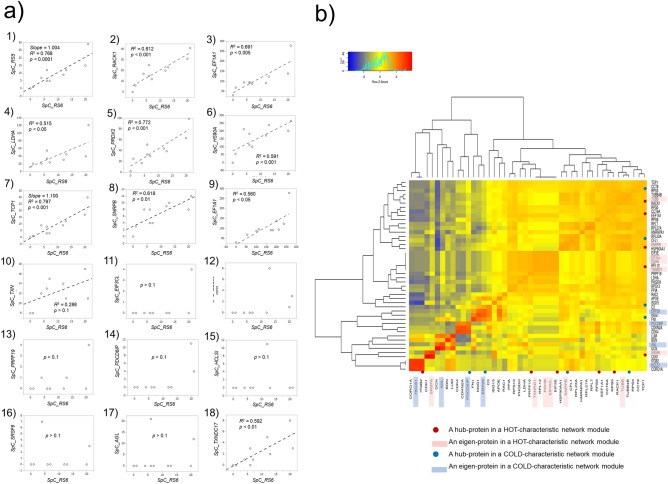
Correlations of representative proteins expressed among all the modules identified for the HOT and COLD traits. (**a**) Spectral count-based semi-quantitative correlations found for eigen- (and/or hub) and other key proteins versus the ribosomal protein S6 (RPS6/RS6), which marks cancer cell growth and proliferation. (**b**) Multivariate correlation analysis (MVA) for the spectral counting-based expression of 46 eigen- (and/or hub-) proteins and other key proteins.

Sonntag et al. performed reverse-phase protein array-based tumor profiling for hormone receptor-positive breast cancer. Through this analysis, caveolin 1 (CAV1), nucleoside diphosphate kinase (NDKA), ribosomal protein S6 (RPS6), and Ki-67 were identified as top candidates for biomarker signatures^29^. 40S RPS6 is a substrate for p70S6 kinase (p70S6K) and a major factor in translational mechanisms, including protein synthesis, cell growth, proliferation, and metabolism. This study revealed that spectral counting-based expression of PRS6 (PS6) was quantitatively correlated with major proteins of the HOT-characteristic four modules, including RACK1, EEF1A1, TXNRD1, and HSP90AA1; nevertheless, it did not correlate with those of the COLD-characteristic four modules (Fig. 4a). Protein co-regulation analysis using ProteomeHD^30^ also exhibited that RACK1 was co-regulated with RPS6 (0.999656), EEF1A1 (0.999593), MCTS1 (0.996626), TXNRD1 (0.9926), HSP90AA1 (0.998816), TCP1 (0.999514), and CCT6 (0.999449) – the numbers in parentheses are percentile scores. These results were confirmed by multivariate correlation analysis (JMP software; SAS Institute, Cary, NC, USA) performed for 46 key proteins including eigen- (or hub-) proteins, demonstrating a close inter-network interaction in a kind of stoichiometric manner among the four following modules: WM2, WM5, WM6, and WM20 (Fig. 4b).

### Master and upstream regulators predicted by ingenuity pathway analysis (IPA)

We conducted an analysis of causal networks and upstream regulators for the identified modules, using the IPA (http://www.ingenuity.com) software^31^. Table 2 lists in brief the top master regulators predicted to be activated or inhibited (|z-value|> 2.0) for the WGCNA modules associated with the HOT lesions. The top 10 master regulators with high values in activation or inhibition score (*z*-score) in causal networks significantly associated with the HOT lesions (WM2, WM5, WM6, WM11, and WM20) and COLD lesions (WM7, WM12, WM18, and WM24), are presented in Tables S1 and S2, together with their participating regulators and target molecules in the datasets.

**Table 2:** A brief list of the top master regulators predicted to be activated or inhibited (|z-value|> 2.0) for the five WGCNA modules which are associated with the HOT lesions.

Module ID (color)	Highly activated master regulator (*z*-score)	Highly inhibited master regulator (*z*-score)
WM2 (lightgreen)	*NMK1/2* (4.123), *MYC* (3.873), MYCN (2.828), and *MLXIPL* (2.646)	chemical drug interventions: 5-fluorouracil (− 2.714), ST1926 (− 2.828), sirolimus (rapamycin) (− 3), SGI-1776 (− 4)
WM5 (cyan)	coagulation factor II thrombin receptor (*F2R/PAR1*) (3.357), integrin beta-4 (*ITGB4*) (3.266), fibroblast growth factor receptor 2 (*FGFR2*) (3.162), and SRSF protein kinase 1 (*SRPK1*) (2.714)	chemical drug interventions: imatinib (− 2.837), emodin (− 2.887), lovastatin (− 2.982), AEE788 (− 3), sitravatinib (− 3.441), and 3D185 (− 4.243)
WM6 (tan)	endothelin-1(*EDN1*) (3.182), NADPH oxidase 4 (*NOX4*) (3.051), apoptosis-antagonizing transcription factor (*AATF*) (2.828), and *Vegf* (group) (2.475)	SD-1008 intervention (− 2.502) and synaptic functional regulator FMR1 (*FMR1*) (− 3)
WM11 (red)	solute carrier family 2, facilitated glucose transporter member 4 (*SLC2A4*) (3.742), bone morphogenetic protein 1 (*Type 1 BMP1*) (3.402), activin receptor-like kinase 1 (*ACTVRL1*) (2.828) and insulin receptor (*INSR*) (2.646)	inositol hexakisphosphate kinase 2 (*IP6K2*) (− 2.746), *mTORC1* (complex) (− 2.921)
WM20 (darkgrey	p21-activated kinase 2 (*PAK2*) (2.333), nuclear factor, erythroid 2 like 2 (*NFE2L2*) (2.236) and 3-hydroxy-3-methylglutaryl-CoA reductase (*HMGCR*) (2.121)	N/A

**Figure 5:**
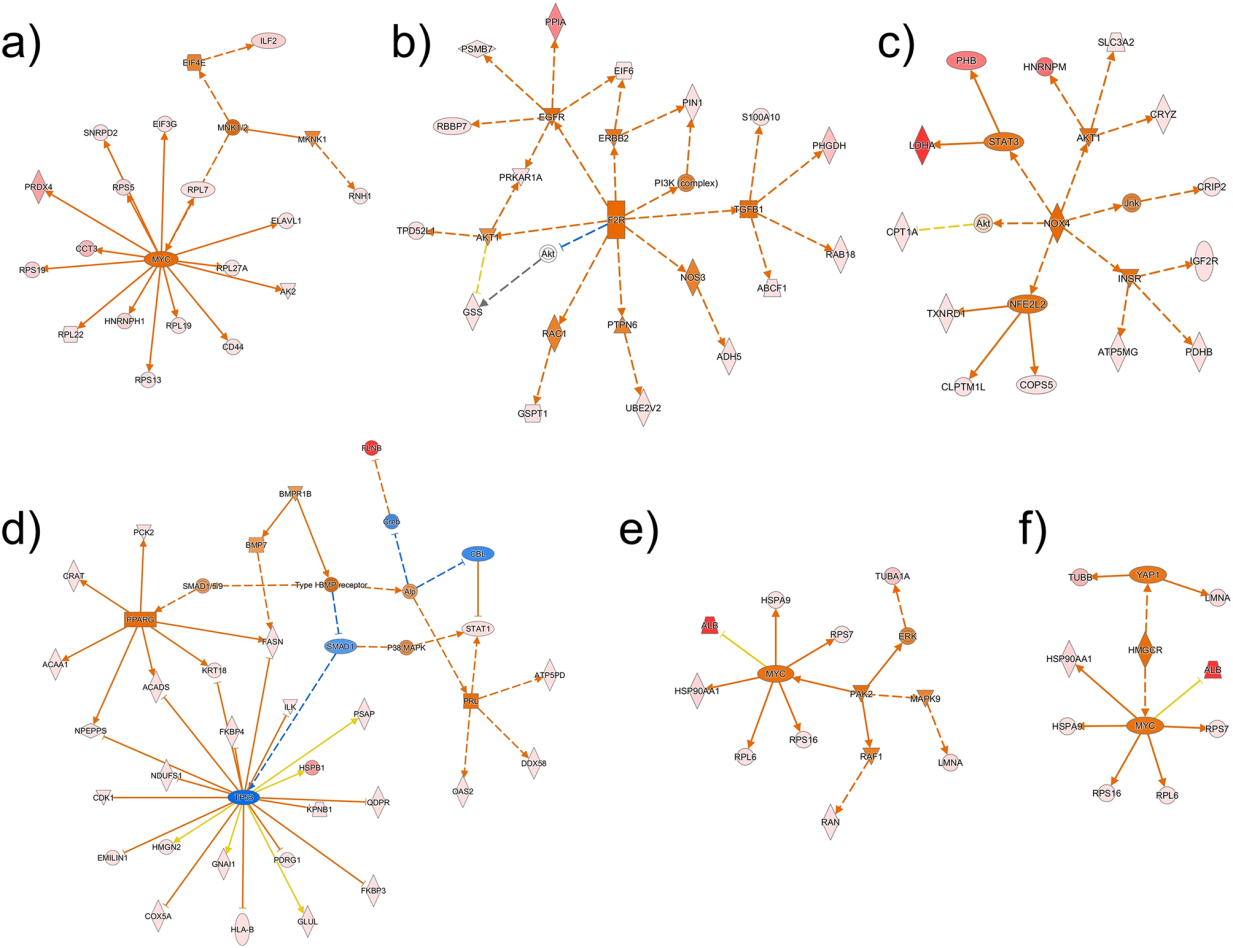
Causal network modules of (**a**) MNK1/2, (**b**) F2R/PAR1, (**c**) NOX4, (**d**) BMP1 receptor, (**e**) PAK2, and (**f**) HMGCR, together with their target molecules in the dataset, which were representatively associated with the HOT trait. Node shapes indicate molecular types: triangle, kinase; square (dashed), growth factor; rectangle (horizontal), ligand-dependent nuclear receptor; rectangle (vertical), ion channel; diamond (vertical), enzyme; diamond (horizontal), peptidase; trapezoid, transporter; oval (horizontal), transcription regulator; oval (vertical), transmembrane receptor; double circle, complex; and circle, other. Red or light red colors indicate highly or moderately increased expression of a mutant protein in the dataset. Orange/light orange and blue/light blue colors indicate the extent of confidence for predicted activation and inhibition, respectively. Lines denote predicted relationships. A solid or dashed line indicates direct or indirect interaction, respectively. Orange, leading to activation; blue, leading to inhibition; yellow, findings inconsistent with the state of a downstream molecule; grey, not predicted effect. BMP1, bone morphogenetic protein 1; F2R/PAR1, coagulation factor II thrombin receptor/ proteinase-activated receptor 1; MNK1/2, MAPK-interacting serine/threonine-protein kinases 1 and 2; NOX4, NADPH oxidase 4; PAK2, p21-activated kinase 2; HMGCR, 3-hydroxy-3-methylglutaryl-CoA reductase.

MAPK-interacting serine/threonine-protein kinases 1 and 2 (MNK1/2), encoded by *MNK1/2* (*MKNK1/2*), were highly activated. MNK1/2 interplays by modulating oncogene eukaryotic initiation factor 4E (EIF4E) between the two major signaling pathways, Ras/MNK and PI3K/AKT/mechanistic/mammalian target of rapamycin (PI3K/AKT/mTOR), which are important in tumorigenesis, oncogenic transformation and progression, and chemoresistance^32, 33^. The PI3K/Akt/mTOR pathway is frequently activated in breast cancer; of note, PIK3CA is the most common mutation in ER-positive breast cancer. *F2R*, also termed proteinase-activated receptor 1 (*PAR1*), encodes a seven-transmembrane G-protein-coupled receptor (GPCR) family member. It was reported that *F2R/PAR1*, as a direct transcriptional target of Twist, enhances the tumorigenic and metastatic capacity of breast cancer cells by suppressing the Hippo pathway and activating epithelial-mesenchymal transition^34^. FGFR2 is a tyrosine-protein kinase that acts as a cell-surface receptor for fibroblast growth factors, playing a crucial role in the regulation of cell proliferation, differentiation, migration, apoptosis, etc. The *FGFR2* locus was reported as the top hit with the risk variants identified repeatedly in genome-wide association studies for ER-positive breast cancer^35^. Genome-wide association studies analyzing 4,398 cases with familial breast cancer causes and 4,316 controls identified five single-nucleotide polymorphisms of *FGFR2* significantly associated with breast cancer^36^. SRPK1 is a serine/arginine-rich protein-specific kinase and one of the core splicing factors^37^; it is highly expressed in more aggressive basal breast cancer. High expression levels of SRPK1 correlated with low metastasis-free survival in patients with ER-positive, but not ER-negative, breast cancer^38^. NOX4 is a ROS-producing NOX protein which has an oncogenic function and produces ROS in the mitochondria^39^. AATF, also termed CHE-1, is involved in transcriptional regulation, cell cycle control, DNA damage responses, and cell death^40^. *SLC2A4*, is also termed glucose transporter type 4, insulin-responsive (GLUT4). High activation of the GLUT4 causal network most likely indicates its estrogen receptor 1 (ESR1)-mediated enhancement^41^. In addition, it may be involved in metabolic reprogramming to oxidative phosphorylation under hypoxia, which increased the activity of the mitochondrial oxidation of pyruvate^42^. Both *BMP1 receptor* and *ACVRL1* are members of the BMP receptors, which may be important in breast cancer promotion and progression. BMP1 receptor comprises BMPR1A and BMPR1B, which belong to transmembrane serine/threonine kinases, the cytokine growth factor transforming growth factor beta (TGFβ) family. Overexpression of BMP receptors exhibits tumor-promoting phenotypes with increased invasion and metastasis, whereas BMP receptors act mostly as tumor suppressors^43^. *ACVRL1* encodes activin receptor-like kinase 1 (ALK1), which is also a type I receptor of the TGFβ superfamily. Since inadequate efficacy of VEGF-targeted agents (including bevacizumab) has been realized, ALK1 has attracted considerable attention as an alternative pathway for the regulation of neo-angiogenesis^44, 45^. *PAK2* encodes serine/threonine-protein kinase PAK2, which is involved in various signaling pathways, including cytoskeleton regulation, cell motility, cell cycle progression, apoptosis, or proliferation. Anti-estrogen resistance in ER-positive breast cancer is associated with activated insulin-like growth factor 1 receptor (IGF1R). Zhang et al. performed a kinome siRNA screening study that identified 10 regulators of IGF1R mediated anti-estrogen. These regulators included the tamoxifen resistance suppressors and inducers, among which *PAK2* is the strongest resistance inducer^46^. *NFE2L2* (or *Nrf2*), encoding the transcription factor Nrf-2 (nuclear factor, erythroid 2 like 2), is a master regulator of antioxidant response^47^. Overexpression of Nrf-2 may contribute to tumorigenesis and chemoresistance by upregulating its target genes^48^. It was also reported that low NFE2L2 mRNA expression levels may be an independent predictor of poor prognosis, especially in ER-positive breast cancer^49^. *HMGCR* encodes 3-hydroxy-3-methylglutaryl-coenzyme A reductase (HMG-CoA reductase), a transmembrane glycoprotein, which is the key enzyme in cholesterol biosynthesis and the target for statin treatment. High mRNA levels of HMGCR together with other genes in the mevalonate pathway were associated with resistance to statin treatment and poor survival in breast cancer^50^. The cholesterol biosynthesis pathway was recently shown to be upregulated in ER-positive breast cancer cell lines resistant to estrogen deprivation. This finding suggested that dysregulation of cholesterol biosynthesis may be a mechanism of endocrine resistance in hormone receptor-positive breast cancer^51^. Representative causal networks associated with the HOT trait are presented in Fig. 5, together with their target molecules in the dataset. Those associated with the COLD trait are presented in Fig. S7.

## Discussion

Our study identified five significant protein-network modules associated with disease mechanisms of this malignant luminal breast carcinomas using WGCNA following in-depth proteomic analysis. The WM2 module centrally involves pathways of ribosome-associated quality control of mRNA and protein including the nonsense-mediated decay. Through these pathways, tumors exploit gene expression for their survival, downregulating the expression of tumor suppressor genes. The WM5 and WM6 modules were both involved in the heat shock response process and angiogenesis caused mainly by oxidative stress and hypoxia. The hub proteins PRDX2 and lactate dehydrogenase A (LDHA) in the WM6 network module are a peroxide scavenger and key enzyme in aerobic glycolysis, respectively. Their function is to protect cancer cells against both oxidative stress and hypoxia. The hub HSP proteins and highly activated *NFE2L2*/*Nrf2* pathway enriched in the WM20 module predominantly participate in pathways of heat shock response and/or oxidative stress. Regarding the WM11 module, CDK1 plays a central role in cell cycle progression and, together with its subnetwork, is involved in oxidative phosphorylation. Collectively, most data-driven protein networks are commonly associated with activities of heat shock response, angiogenesis, and cancer cell survival. A semi-quantitative correlation of key-protein expression, protein co-regulation analysis using ProteomeHD, and multivariate correlation analysis suggested co-regulations via network-network interaction among the four key modules characteristic to the HOT lesions.

**Figure 6:**
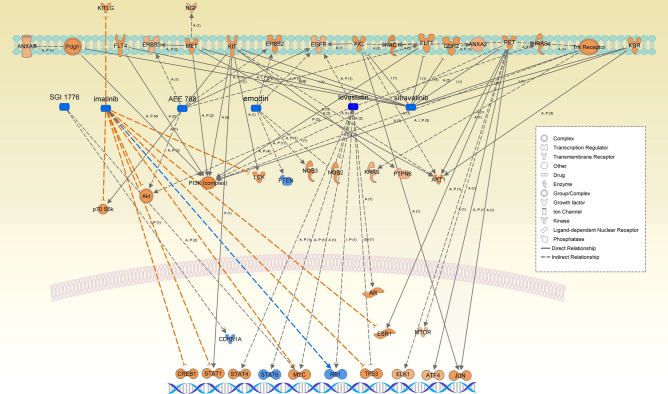
The causal networks of chemical drug interventions (SGI-1776, imatinib, emodin, lovastatin, AEE788, and sitravatinib) are predicted to be highly inhibited in the WM5 (cyan) network module.

Highly activated master and upstream regulators of causal networks predicted to the five data-driven protein networks were mostly characteristic to ER-positive breast cancer, as well as associated with oncogenic transformation, resistance to chemotherapy, and endocrine therapy. The integrative networks constructed from the representative master and participating regulators predicted for the HOT-characteristic WGCNA modules exhibited a predominant activation of ESR1, MYC, nuclear receptor 4A1 (NR4A1), and NFE2L2 (Fig. S8). Interestingly, the top causal networks predicted for the WM2 and WM5 modules listed highly inhibited interventions by numerous chemical inhibitors, indicating the involvement of potential therapeutic targets. The WM2 module included 5-fluorouracil, sirolimus (rapamycin), and SGI-1776, while the WM5 module included imatinib, emodin, lovastatin, AEE788, and sitravatinib (Fig. 6).

The limitation of this study is the number of patients examined, which was attributed to only these 5 cases of Ki-67 values at HOT > 80-% available in our hospital, corresponding to 3.8% (5/133) of the total Luminal-B like cases. We plan to verify/validate the results of this study by using a larger sample size of the external cohort being accumulated in the future.

In conclusion, the WGCNA combined with ORA-based protein screening was successfully applied to clinical proteomic datasets. Our results could identify key data-driven networks and their upstream regulators characterizing the HOT lesions. The limitation of this study is the small number of patients analyzed. We are planning a larger cohort study of patient-derived samples, including genomic alteration analysis to investigate core data-driven proteogenomic networks. This approach will provide clinically important information on proteogenomic landscapes of ER+HER2–Ki-67high malignant luminal breast cancer.

## Methods

### FFPE tissue specimens and sample preparation

A total of 671 patients underwent surgical breast cancer resection at the Tokyo Medical University Hachioji Medical Centre between 2015 and 2019. Pathological specimens were independently reviewed by three pathologists (MW, ES, and HH) to confirm that they satisfied the 2015 WHO classification of breast tumors (histological criteria)^52^. Only 21 tumors were histologically confirmed as ER+HER2–Ki-67high (> 70%) luminal B breast cancers. Of those, five cases with high Ki-67 (> 80%) were selected for investigation in this study. FFPE tumor tissue blocks from the corresponding five surgical specimens were obtained without patient identifiers from the Tokyo Medical University Hospital and Hachioji Medical Centre. Informed consent was provided by all participating patients, and the study protocol was approved by the institutional review board of the Tokyo Medical University Hospital (approval no. H-266). The study was conducted in accordance with the tenets of the Declaration of Helsinki.

The terms HOT and COLD are defined as follows: HOT spot, the highest density of Ki-67-positive cells in the lesion (i.e., area with the highest Ki-67 value); COLD spot, the lowest density of Ki-67-positive cells (i.e., area with the lowest Ki-67 value). For tissue microdissection, sections (thickness: 10 μm) from the FFPE tumor blocks were cut onto DIRECTOR slides (OncoPlex Diagnostics Inc., Rockville, MD, USA). Prior to dissection, the sections were de-paraffinized and stained only with hematoxylin using standard histological methods. Microdissection was performed using a Leica LMD7 Microdissection Microscope (Leica, Wetzlar, Germany) (see Fig. S2). Typically, a total area of 5 mm^2^ with approximately 19,000 tumor cells was transferred from the FFPE sections via laser dissection directly into the cap of a 200-μL low-binding tube. Proteins were extracted and digested with trypsin using Liquid Tissue MS Protein Prep kits (OncoPlex Diagnostics Inc.) according to the instructions provided by the manufacturer^53^. The procedures were previously described^6, 7^.

Briefly, dried microdissection pellets were suspended in 20 μL of Liquid Tissue buffer, heated at 95 °C for 90 min, and then cooled on ice, at which point 0.1 μg of trypsin was added to each tube. The tubes were then incubated at 37 °C for 18 h. The digested samples were dried and resuspended in 50 μL of 2% acetonitrile aqueous solution containing 0.1% TFA. Finally, the digested samples were frozen at − 80 °C until further processing.

### MS-based proteomic analysis

The digested individual samples (5 μL for a single run) were analyzed in triplicate by liquid chromatography-tandem mass spectrometry (LC–MS/MS) analysis using reverse-phase LC interfaced with a Q Exactive Orbitrap mass spectrometer (Thermo Fisher Scientific, Bremen, Germany) via a nano-ESI device Dream-Spray (AMR Inc., Tokyo, Japan). The LC system consisted of an Ultimate 3000 HPLC System (Thermo Fisher Scientific), a trap-cartridge L-column (0.3 mm × 5.0 mm, CERI, Tokyo, Japan), and a capillary separation column (Zaplous column alpha-PepC18, 3 μm, 12 nm, 0.1 mm × 150 mm, AMR Inc.). An auto-sampler (HTC-PAL, CTC Analytics, Zwingen, Switzerland) loaded an aliquot of samples into the trap, which was then washed with solvent A (2% acetonitrile aqueous solution containing 0.1% trifluoroacetic acid) to concentrate the peptides in the trap and desalt them. Subsequently, the trap was connected in series to the separation column, and the peptides were eluted from the whole column with 0.1% formic acid aqueous solution and acetonitrile by linear 5–40% acetonitrile concentration gradient over 90 min at a flow-rate of 500 nL min^−1^. The mass spectrometer was operated in data-dependent acquisition mode.

All LC–MS/MS data were acquired using Xcalibur, version 4.0.27.19 (Thermo Fisher Scientific) in high-resolution data-driven analysis (DDA) mode, with the survey scan (MS in the mass range m/z 400–1600) acquired in the Orbitrap at 70,000 resolution (at m/z 200) in profile mode. The top ten most intense peaks from the survey scan were selected for fragmentation with higher-energy collisional dissociation with a normalized collision energy of 27 and an isolation window of m/z 1.6. The dynamic exclusion time for precursor ions selected for MS/MS fragmentation was 10 s, and the automatic gain control target values for MS and MS/MS were 1 × 10^6^ and 1 × 10^5^, respectively.

Peptide sequence matching was performed using the MASCOT software ver. 2.6.0 (http://www.matrixscience.com) against the UniProt Homo sapiens database downloaded in January 2017. A target-decoy search strategy was employed for increased confidence in protein identifications^54^. This search considered tryptic peptide candidates, and the formylation of lysine and oxidation of methionine were considered as variable modifications. The MASCOT search engine considered a precursor mass tolerance of 10 ppm and a fragment bin tolerance of 0.02. The validity of the peptide spectrum matches was assessed using the MASCOT software. All identification results were reported with < 1% FDR, both at the peptide and protein levels.

The expression levels of identified proteins were assessed by spectral count-based protein quantification (Fig. S4). Fold changes in protein expression in the base 2 logarithmic scale (*R*_*SC*_)^55^ and normalized spectral abundance factors (*NSAF*)^56^ representing relative abundances of expressed proteins were calculated using the spectral count (*SpC*)—that is the number of MS/MS spectra assigned to each protein.

### Weighted correlation network analysis

Weighted-gene co-expression network analysis (WGCNA)^8^ was used to identify systems-level differences in the protein expression pattern of the HOT and COLD spots in malignant luminal breast tissues. The similarity in protein expression patterns for all protein pairs was calculated according to their pairwise Pearson’s correlation coefficient, i.e., the similarity between proteins i and j was defined as (1 − *r*_*i,j*_)/2, where *r*_*i,j*_ is the Pearson’s correlation coefficient of the protein expression patterns between these two proteins. We performed the network topology analysis for various soft-thresholding powers ranging from 1 to 100 to choose an optimal value to balance between independence and mean connectivity. The power had been set to 10, under which the network reaching to the scale-free topology. By implying a soft thresholding parameter, the weighted gene expression network emphasizes highly correlated protein pairs and filters low correlations. This reduces the noise of correlation in the adjacency matrix until the network resembles a scale-free graph. Next, to measure the connection strength between all protein pairs, the topological overlap measure (TOM) was calculated from the adjacency matrix. TOM dissimilarity matrix (1-TOM) was subsequently used to perform average linkage hierarchal clustering, which generated a protein clustering tree with modules corresponding to the branches of the tree. Dynamic tree cutting was used to trim the branches and identify protein modules.

Modules were summarized by the first principal component referred to as eigen-protein in the text. Module membership, defined as the correlation between the protein expression profile and the module eigen-protein, was measured with values ranging 0–1; 0 represents a gene that is not part of the module, while 1 represents high connectivity to the module. Subsequently, the module-trait association was determined using the correlation between the module eigen-protein and the two clinical traits (HOT and COLD). WGCNA analysis was performed using the WGCNA R-package^8^, implemented as a gadget in the GARUDA PLATFORM (The Systems Biology Institute, Tokyo, Japan).

### PPI network construction

We used the Search Tool for the Retrieval of Interacting Genes/Proteins (STRING) database (version 10.5) to construct a protein interaction network for a protein module^15^. STRING networks were calculated under the criteria for linkage only with experiments, databases, text mining, and co-expression with the default settings i.e., medium confidence score: 0.400, network depth: 0 interactions. Functional enrichment results were obtained for canonical pathways under *p* < 0.05. Proteins in a protein module were mapped in the protein interaction network from the STRING database, to produce the results of the enrichment analysis on the biological process (GO) and Reactome pathways.

A hub protein refers to a “highly connected protein.” The proteins inside co-expression modules exhibit high connectivity, and the proteins within the same module may play similar roles. The PPI networks were reconstructed using the Cytoscape software (version 3.7.1.), followed by importation of the results obtained from the STRING PPI network analysis of eigen-proteins in each module. We identified hub proteins in each module according to their intra-modular connectivity and correlation with module eigen-proteins. The top 20 high-degree proteins were identified using the *cytoHubba plugin*^16^. The three top-ranked genes in each module were considered to be hub proteins.

### Causal network analysis by IPA

Upstream regulators and causal networks were predicted using the IPA software^31^. Protein expression data (quantile-normalized for selected modules) were used as input datasets. Causal networks (*p* < 0.05) were predicted from the WGCNA network modules significantly associated with the two clinical traits (HOT and COLD), where their activation or inhibition was defined by *z*-values > 2.0 or <  − 2.0, respectively.

## Supplementary Information

Supplementary Information.

## Data Availability

The unfiltered MS datasets generated and analyzed in this study have been deposited in the in the ProteomeXchange (http://proteomecentral.proteomexchange.org) and jPOST with the dataset identifiers PXD021912 and JPST000981, respectively.
