# Supplementary material for: Protein co-expression networks identified from HOT lesions of ER+HER2–Ki-67high luminal breast carcinomas
Source: Sci Rep. 2021 Jan 18;11:1705. doi: 10.1038/s41598-021-81509-9 (PMC7814020; doi:10.1038/s41598-021-81509-9)
Supplement: Supplementary file 1 — Supplementary Information. [file 41598_2021_81509_MOESM1_ESM.pdf]

Supplementary Information File 1

Protein Co-expression Networks Identified from HOT Lesions of ER +HER2−Ki-67high Luminal Breast Carcinomas

Kimito Yamada, Toshihide Nishimura*, Midori Wakiya, Eiichi Satoh, Tetsuya Fukuda, Kengo Amaya, Yasuhiko Bando, Hiroshi Hirano, Takashi Ishikawa

Figure S1. Breast cancer surgical cases at at Tokyo Medical University Hospital Hachioji Medical Center during 2015 – 2019.

(a) Subtypes of 671 breast cancer patients received surgical operation, in which the luminal A and B subtypes were about 58% and 20 %, respectively.

(b) The Kaplan-Meier (KM) plots of recurrent free survivals for luminal A and B cases, showing that the high risk group was luminal B cases and the low risk group was luminal A cases with the log-rank test p-value < 0.0000001.

(c) The KM plots of recurrent free survivals for luminal B cases among groups with high Ki-67 values (>50%) or with low Ki-67 values (30 – 49 %). which demonstrated that the high risk group was high Ki-67 cases and the low risk group was low Ki-67 cases, respectively, with the log-rank test p-value < 0.005.

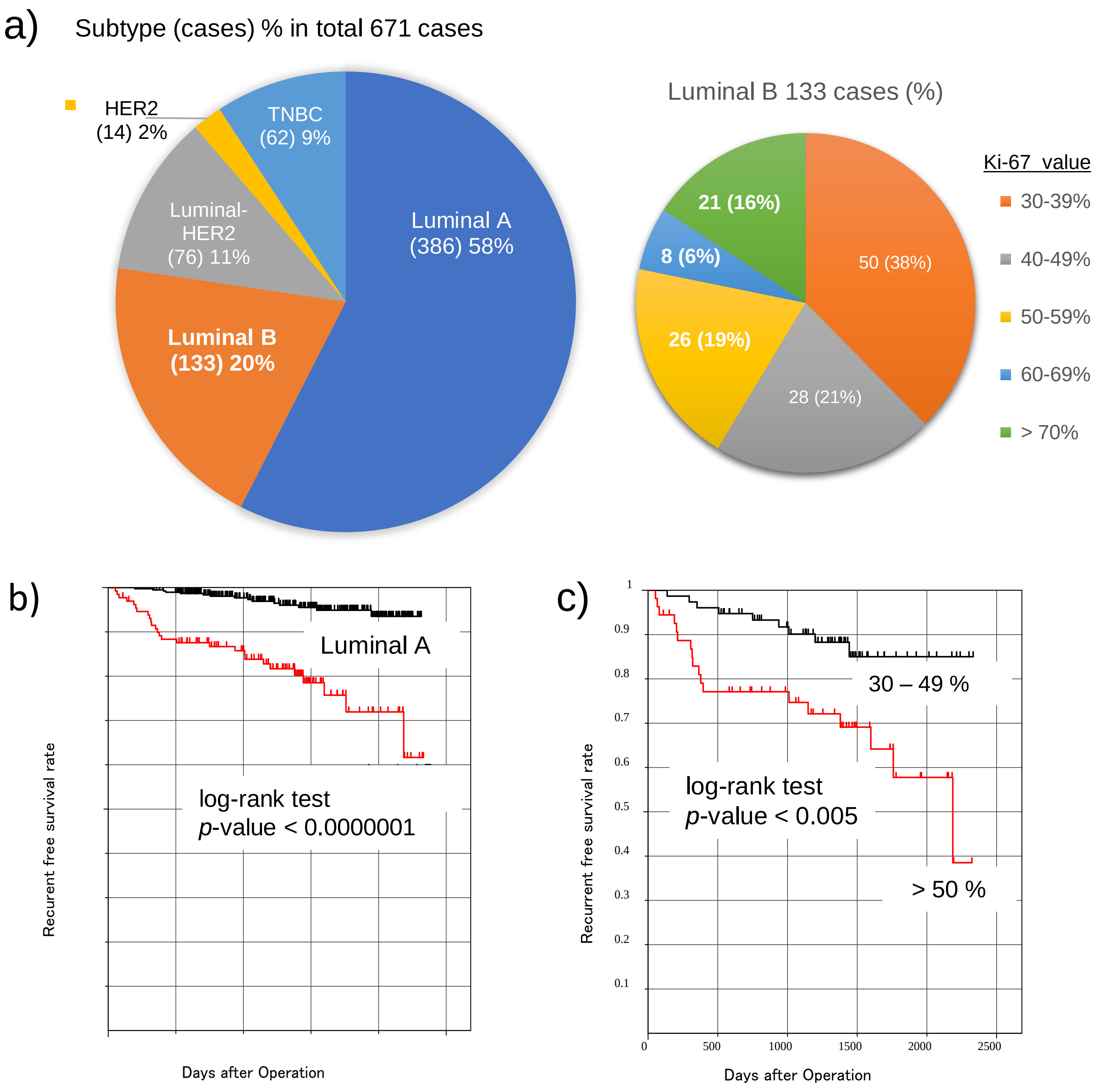

Figure S2. Laser micro-dissection (LMD) of targeted HOT and COLD lesions from the luminal B breast carcinomas. (**a**) Hematoxylin and eosin (HE) staining, (**b**) Ki-67 immunohistological staining, (**c**) Befor LMD, and (**d**) After LMD.

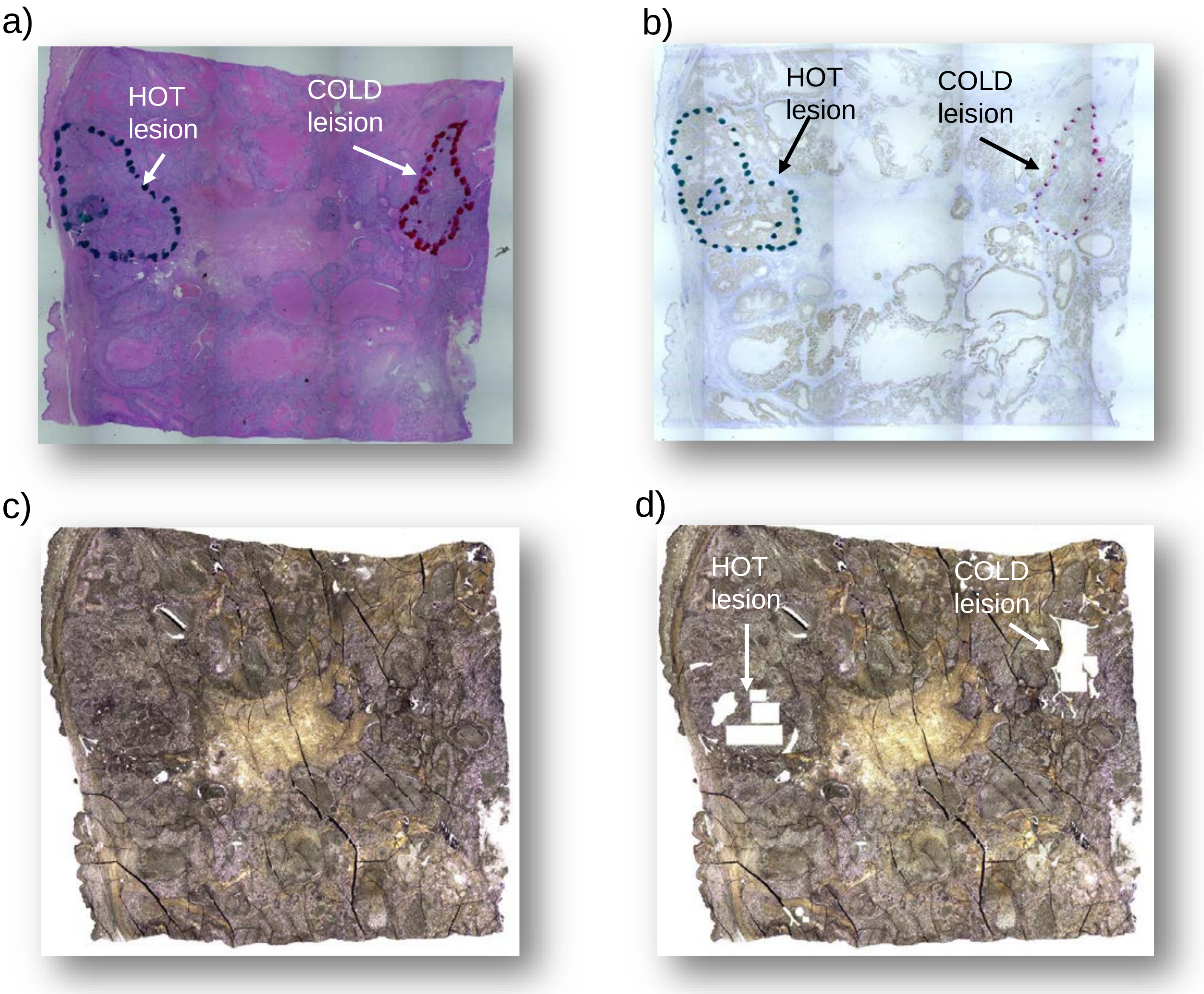

Figure S3. Profiles of proteins identified to the HOT and COLD lesions of the ER+HER2-Ki67high luminal breast carcinoma subtype.

a)

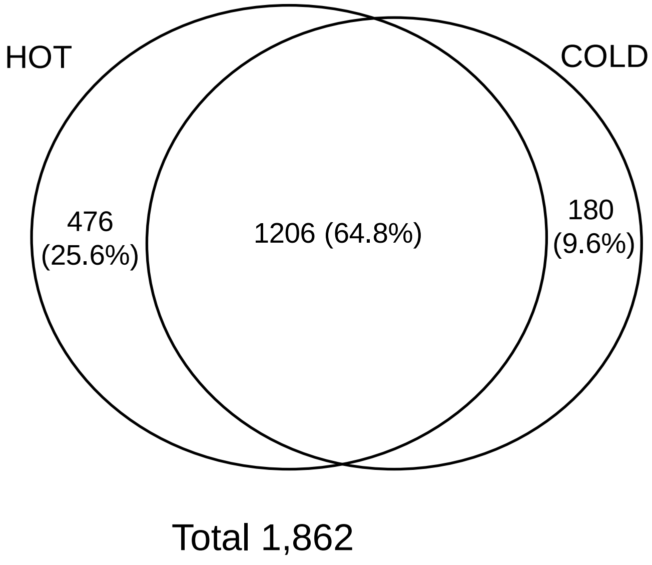

Targeted microdissected lesions	# of idenfied proteins	Total spectral counts (*SpCs*)
HOT	1682	96469
COLD	1386	87851
	Average	92160
	SD	4.7%

b)

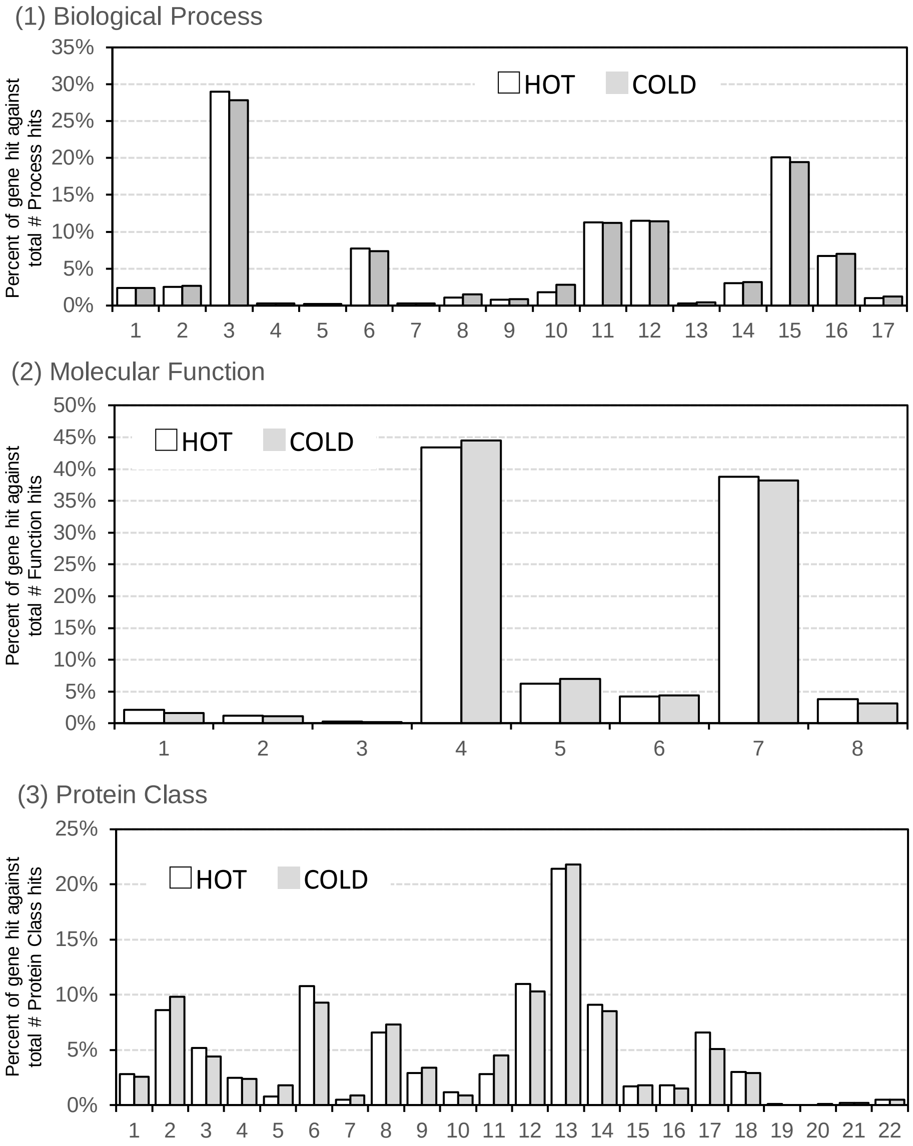

Figure S4. R_{SC} values between HOT and COLD calculated for proteins identified (X-axis). Proteins upregurlated with twice fold changes for HOT ($R_{SC} \leq -1$) and COLD ($R_{SC} \geq 1$) are denoted.

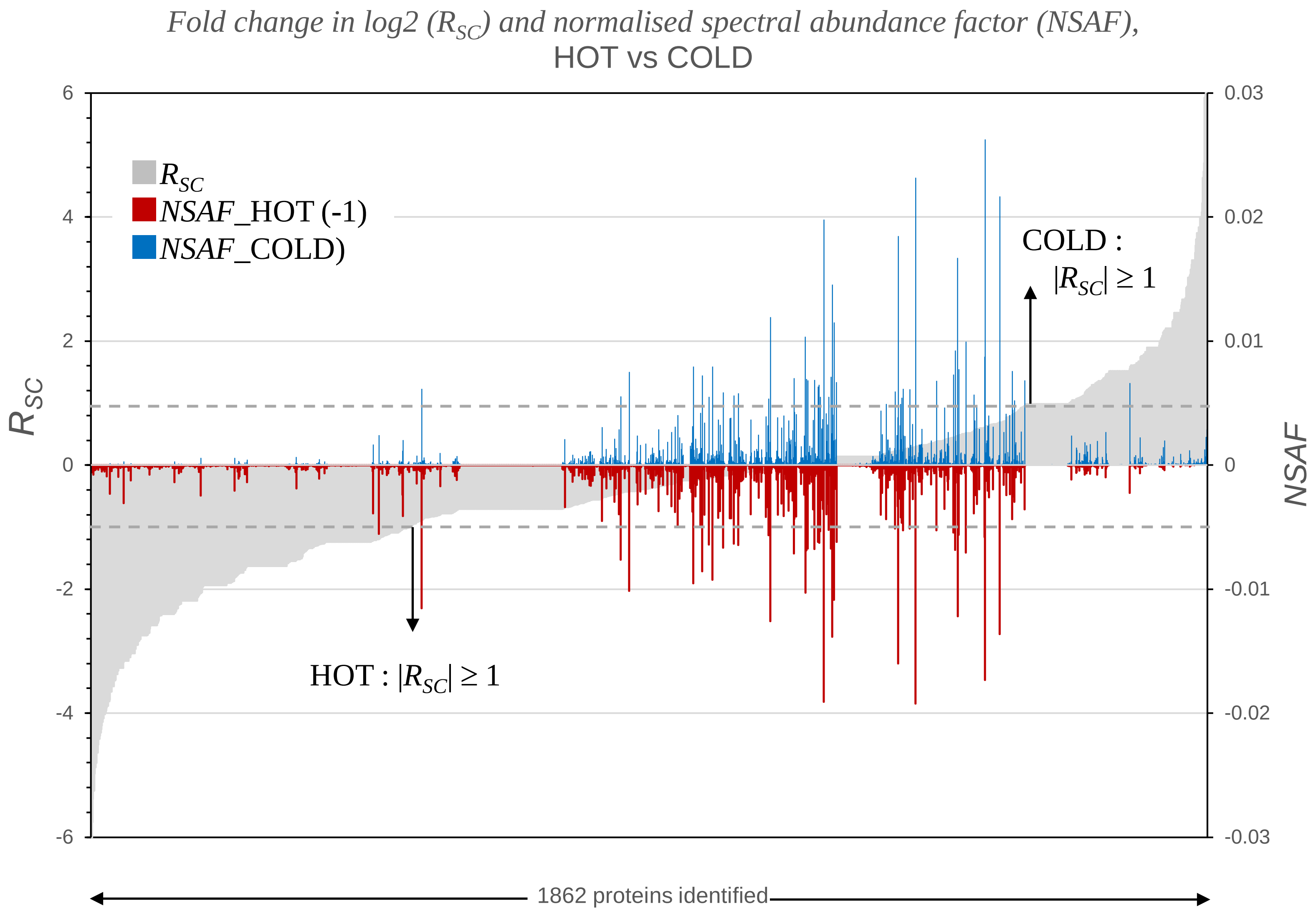

Figure S5. Module trait relationship between the HOT and COLD trait, and Module membership (MM) vs. gene significance (GS)

Module No.	Module colour	*Module Trait Correlation* HOT	COLD	*p*-value	*No. of genes*	*Module Membership vs. Gene Significance* *Corr.*	*p*-value
WM1	darkorange	0.303	-0.303	0.3951	13	-0.43	0.14
WM2	lightgreen	0.412	-0.412	0.2365	31	0.23	0.21
WM3	darkred	-0.288	0.288	0.4196	18	0.45	0.061
WM4	turquoise	0.218	-0.218	0.5447	283	-0.024	0.69
WM5	cyan	0.429	-0.429	0.2165	53	0.96	7.4E-30
WM6	tan	0.392	-0.392	0.2623	57	0.59	1.4E-06
WM7	green	-0.331	0.331	0.3499	112	0.29	0.0019
WM8	midnightblue	-0.145	0.145	0.6886	48	-0.11	0.46
WM9	pink	0.007	-0.007	0.9853	78	-0.51	1.8E-06
WM10	darkturquois e	-0.29	0.29	0.4163	16	0.15	0.58
WM11	red	0.354	-0.354	0.3153	102	0.47	6.2E-07
WM12	greenyellow	-0.297	0.297	0.4048	60	0.0086	0.51
WM13	blue	0.29	-0.29	0.417	171	0.33	0.00001
WM14	skyblue	-0.261	0.261	0.4672	11	0.043	0.9
WM15	magenta	0.045	-0.045	0.9014	70	-0.47	0.00004
WM16	royalblue	-0.191	0.191	0.5964	25	0.094	0.65
WM17	salmon	-0.286	0.286	0.4236	54	0.42	0.0016
WM18	black	-0.367	0.367	0.2971	81	0.46	0.000016
WM19	lightcyan	-0.147	0.147	0.6853	42	-0.014	0.93
WM20	darkgrey	0.412	-0.412	0.2372	16	0.44	0.088
WM21	white	-0.129	0.129	0.722	13	-0.4	0.18
WM22	purple	0.104	-0.104	0.7751	69	-0.75	1.2E-13
WM23	yellow	0.315	-0.315	0.3751	146	0.37	4.3E-06
WM24	brown	-0.311	0.311	0.3818	166	0.26	0.00072
WM25	grey60	0.267	-0.267	0.4559	36	-0.096	0.58
WM26	orange	0.005	-0.005	0.9882	15	-0.76	0.001
WM27	darkgreen	0.086	-0.086	0.8127	18	-0.14	0.58
WM28	lightyellow	-0.081	0.081	0.8229	27	-0.37	0.057

Corr .
0.43
0
-0.43

Figure S6. Data-driven protein co-expression networks identified and pathway enrichment results for the COLD trait.

a)

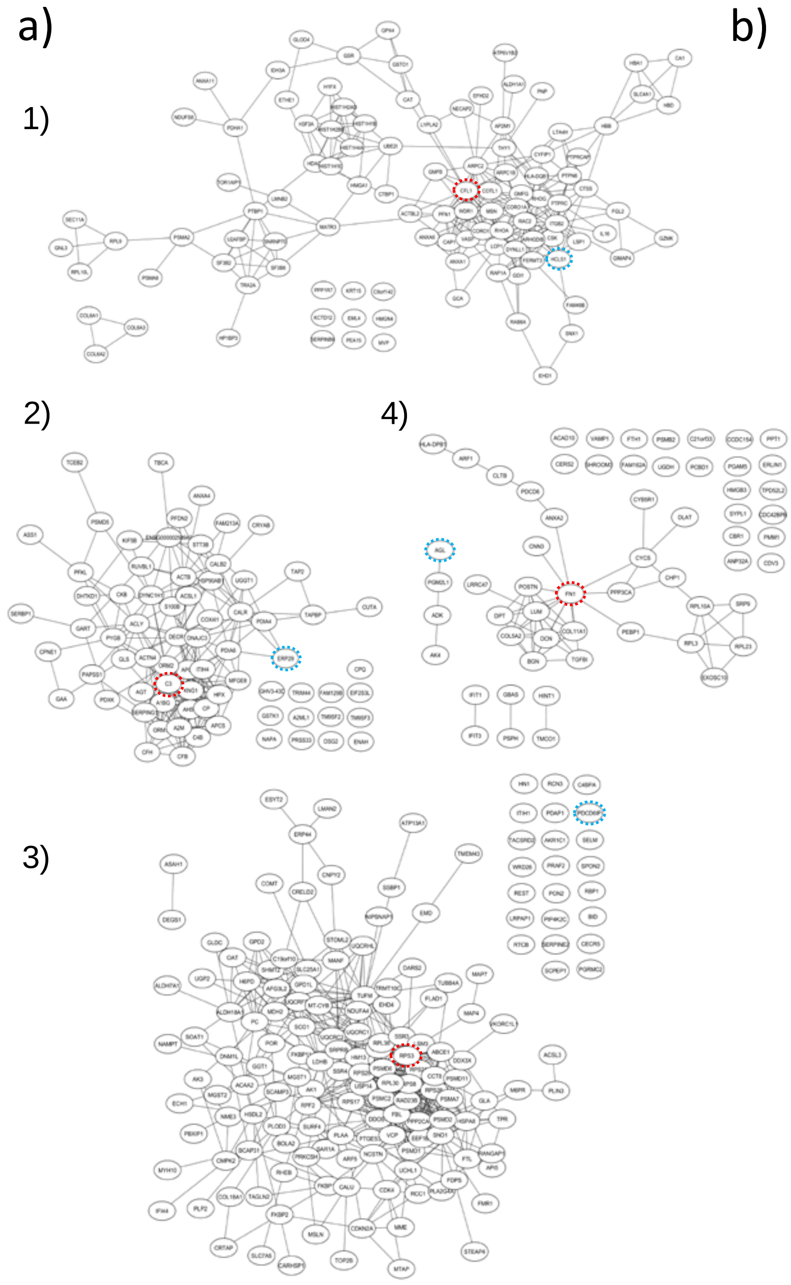

b)

Module ID (coloe)	Biological process (GO)					Reactome pathways				
	#pathway ID	pathway description	observed gene count	background gene count	false discovery rate	#term ID	Pathway description	observed gene count	background gene count	false discovery rate
WM7 (green)	GO:0002252	immune effector process	28	927	4.29E-10	HSA-168249	Innate Immune System	21	1012	0.000000038
	GO:0002443	leukocyte mediated immunity	24	632	4.29E-10	HSA-168256	Immune System	28	1925	0.000000038
	GO:0002444	myeloid leukocyte mediated immunity	22	519	4.29E-10	HSA-114608	Platelet degranulation	9	125	0.000000231
	GO:0002446	neutrophil mediated immunity	21	498	6.40E-10	HSA-6798695	Neutrophil degranulation	14	471	0.00000024
	GO:0006887	exocytosis	25	774	6.40E-10	HSA-8957275	Post-translational protein phosphorylation	8	106	0.000000696
	GO:0043299	leukocyte degranulation	21	507	6.40E-10	HSA-381426	Regulation of Insulin-like Growth Factor (IGF) transport and uptake by Insulin-like Growth Factor Binding Proteins (IGFBPs)	8	123	0.00000178
	GO:0045055	regulated exocytosis	24	691	6.40E-10	HSA-977606	Regulation of Complement cascade	5	47	0.0000658
	GO:0043312	neutrophil degranulation	20	485	1.75E-09	HSA-392499	Metabolism of proteins	22	1948	0.0000659
	GO:0002366	leukocyte activation involved in immune response	22	616	1.80E-09	HSA-109582	Hemostasis	12	601	0.0000954
	GO:0016192	vesicle-mediated transport	35	1699	1.80E-09	HSA-174577	Activation of C3 and C5	3	7	0.00019
WM12 (greenyellow)	GO:0030198	extracellular matrix organization	10	296	0.0000423	HSA-1430728	Metabolism	22	2032	0.0000162
	GO:0030199	collagen fibril organization	5	39	0.00012	HSA-1643685	Disease	11	1018	0.0161
	GO:0044281	small molecule metabolic process	19	1779	0.00026	HSA-3000178	ECM proteoglycans	4	75	0.0161
	GO:1901135	carbohydrate derivative metabolic process	15	1083	0.00026	HSA-71291	Metabolism of amino acids and derivatives	7	354	0.0161
	GO:0009057	macromolecule catabolic process	14	970	0.00028	HSA-71387	Metabolism of carbohydrates	6	266	0.0161
	GO:1901566	organonitrogen compound biosynthetic process	16	1370	0.00054	HSA-3560782	Diseases associated with glycosaminoglycan metabolism	3	40	0.0168
	GO:1901575	organic substance catabolic process	17	1609	0.00081	HSA-1630316	Glycosaminoglycan metabolism	4	122	0.0183
	GO:0044248	cellular catabolic process	17	1646	0.00098	HSA-1799339	SRP-dependent cotranslational protein targeting to membrane	4	109	0.0183
	GO:0009056	catabolic process	18	1859	0.001	HSA-3595172	Defective CHST3 causes SEDCJD	2	8	0.0183
	GO:0017144	drug metabolic process	10	622	0.0027	HSA-3595174	Defective CHST14 causes EDS, musculocontractural type	2	8	0.0183
WM18 (black)	GO:0032940	secretion by cell	24	959	1.22E-10	HSA-168249	Innate Immune System	21	1012	0.000000038
	GO:0016192	vesicle-mediated transport	29	1699	6.22E-10	HSA-168256	Immune System	28	1925	0.000000038
	GO:0045055	regulated exocytosis	19	691	4.70E-09	HSA-114608	Platelet degranulation	9	125	0.000000231
	GO:0006955	immune response	26	1560	1.51E-08	HSA-6798695	Neutrophil degranulation	14	471	0.00000024
	GO:0002252	immune effector process	19	927	0.000000351	HSA-8957275	Post-translational protein phosphorylation	8	106	0.000000696
	GO:0002443	leukocyte mediated immunity	16	632	0.000000432	HSA-381426	Regulation of Insulin-like Growth Factor (IGF) transport and uptake by Insulin-like Growth Factor Binding Proteins (IGFBPs)	8	123	0.00000178
	GO:0006810	transport	39	4130	0.000000432	HSA-977606	Regulation of Complement cascade	5	47	0.0000658
	GO:0002376	immune system process	29	2370	0.000000495	HSA-392499	Metabolism of proteins	22	1948	0.0000659
	GO:0002576	platelet degranulation	9	129	0.000000495	HSA-109582	Hemostasis	12	601	0.0000954
	GO:0043312	neutrophil degranulation	14	485	0.000000669	HSA-174577	Activation of C3 and C5	3	7	0.00019
WM24 (brown)	GO:0044281	small molecule metabolic process	52	1779	1.50E-12	HSA-1430728	Metabolism	58	2032	9.11E-15
	GO:1901564	organonitrogen compound metabolic process	88	5281	6.19E-10	HSA-1799339	SRP-dependent cotranslational protein targeting to membrane	14	109	5.58E-10
	GO:0006810	transport	75	4130	1.54E-09	HSA-71291	Metabolism of amino acids and derivatives	19	354	5.68E-08
	GO:0051234	establishment of localization	76	4248	1.56E-09	HSA-9010553	Regulation of expression of SLITs and ROBOs	14	164	5.68E-08
	GO:0051179	localization	83	5233	3.59E-08	HSA-72766	Translation	17	288	8.51E-08
	GO:0043603	cellular amide metabolic process	27	732	7.16E-08	HSA-5663205	Infectious disease	18	363	0.000000303
	GO:0019752	carboxylic acid metabolic process	29	854	7.67E-08	HSA-156842	Eukaryotic Translation Elongation	10	90	0.000000822
	GO:0017144	drug metabolic process	24	622	0.000000269	HSA-392499	Metabolism of proteins	41	1948	0.00000176
	GO:0044248	cellular catabolic process	40	1646	0.000000269	HSA-6798695	Neutrophil degranulation	19	471	0.00000176
	GO:0006613	cotranslational protein targeting to membrane	11	97	0.000000458	HSA-975957	Nonsense Mediated Decay (NMD) enhanced by the Exon Junction Complex (EJC)	10	112	0.00000408

Figure S7. Causal network modules representatively associated with the COLD trait. (a) TSC22D1, (b) MAPK3, (c) IGF receptor, and (d) SMAD5.

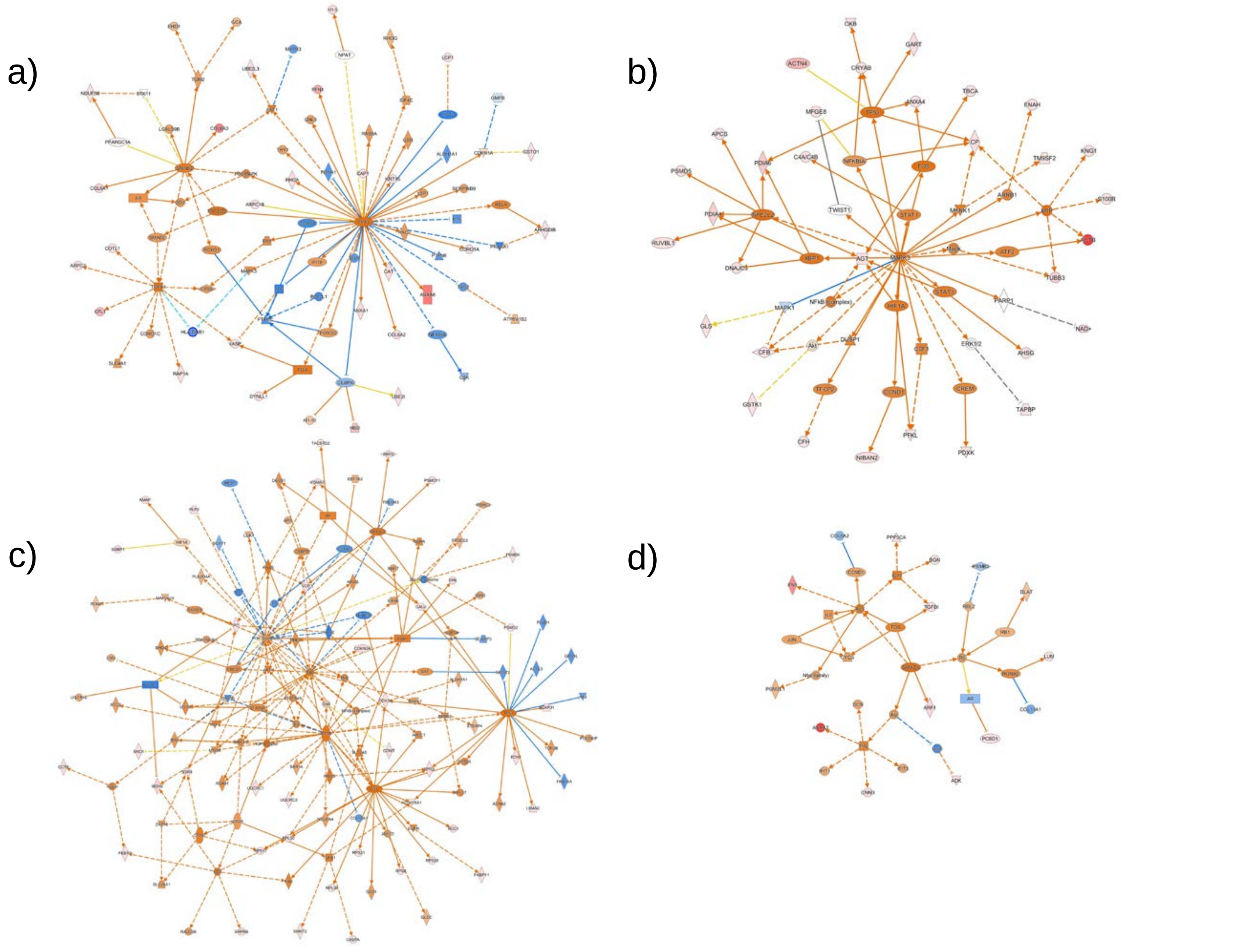

Figure S8. The integrative networks of representative master and participating upstream regulators predicted for the WGCNA network modules characteristic to the HOT lesions.

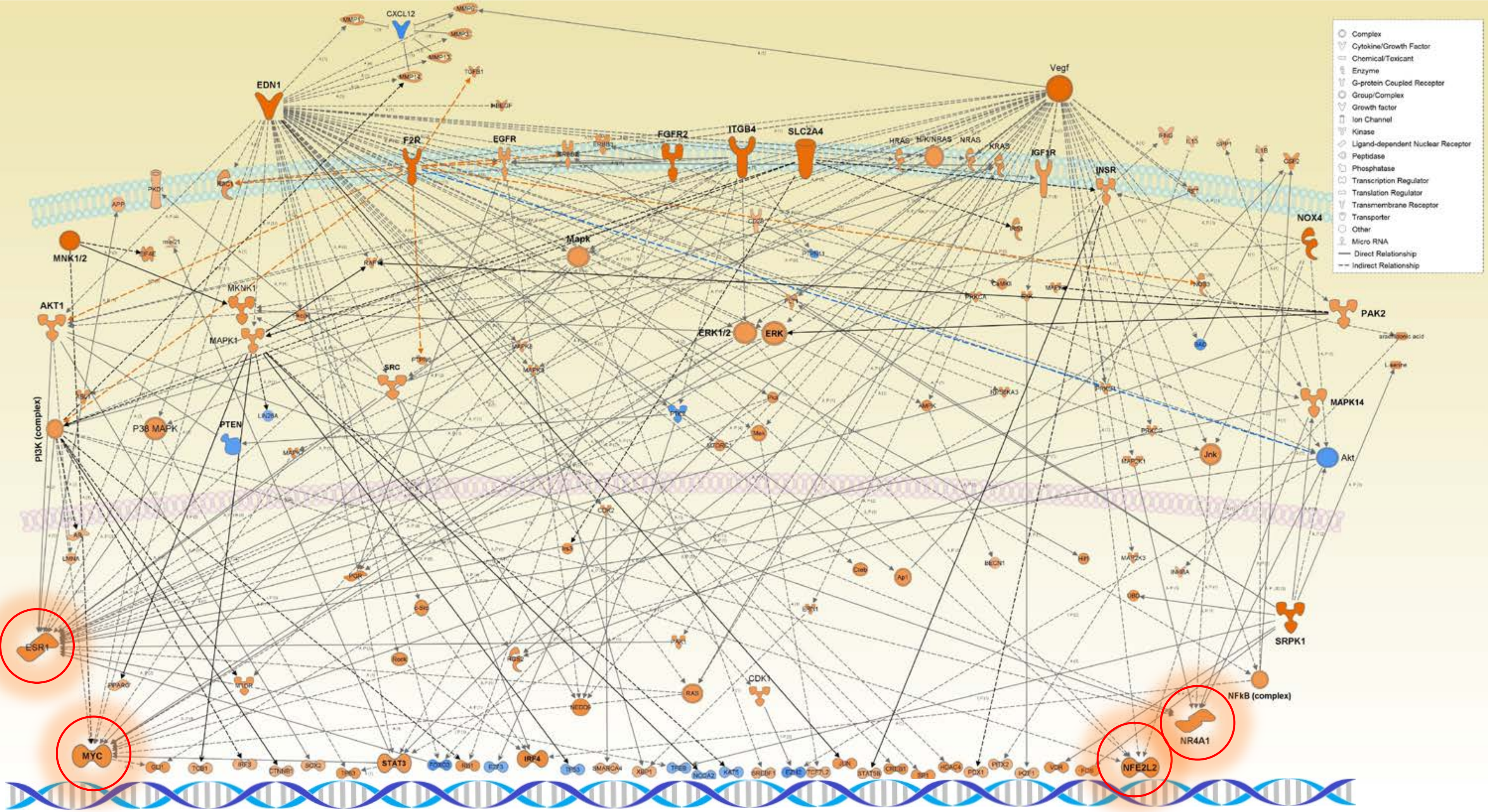

Table S1. The top 10 master regulators and participating regulators of the network modules significant to the HOT lesions (WM2, WM5, WM6, WM11, and WM20), which were predicted to have high values in activation or inhibition score (z-score) using the Ingenuity Pathway Analysis (IPA). Chemical drugs are indicated in red letter.

Module No. (color)	Master Regulator	Molecule Type	Participating regulators	Depth	Predicted Activation	Activation z-score	p-value of overlap	Network bias-corrected p-value	Target molecules in dataset
WM2 (lightgreen)									
	MNK1/2	group	EIF4E,MKNK1,MNK1/2,MYC	2	Activated	4.123	1.8E-14	0.0001	AK2,CCT3,CD44,EIF3G,ELAVL1,HNRNPH1,ILF2,PRDX4,RNH1,RPL19,RPL22,RPL27A,RPL7,RPS13,RPS19,RPS5,SNRPD2
	MYC	transcription regulator	MYC	1	Activated	3.873	7.25E-13	0.0001	AK2,CCT3,CD44,EIF3G,ELAVL1,HNRNPH1,PRDX4,RPL19,RPL22,RPL27A,RPL7,RPS13,RPS19,RPS5,SNRPD2
	MYCN	transcription regulator	MYCN	1	Activated	2.828	1.73E-09	0.0001	RPL19,RPL22,RPL23A,RPL27A,RPL7,RPS13,RPS19,RPS5
	MLXIPL	transcription regulator	MLXIPL	1	Activated	2.646	1.88E-10	0.0001	RPL19,RPL22,RPL27A,RPL7,RPS13,RPS19,RPS5
	5-fluorouracil	chemical drug	5-fluorouracil	1	Inhibited	-2.714	1.34E-13	0.0001	ATP5PB,CCT3,EIF3G,ILF2,MDH1,RPL19,RPL27A,RPL7,RPS13,SNRPB,SNRPD2
	ST1926	chemical drug	ST1926	1	Inhibited	-2.828	4.69E-11	0.0001	AK2,CCT3,EIF3G,ILF2,RPL19,SNRPB,SNRPD2,SRSF3
	sirolimus	chemical drug	sirolimus (Rapamycin)	1	Inhibited	-3	2.78E-08	0.0001	GSTP1,RACK1,RPL19,RPL27A,RPS13,RPS19,RPS5,SNRPD2,SRSF3
	PFDN5	transcription regulator	MYC,PFDN5,TP73	2	Inhibited	-3.153	9.32E-14	0.0001	AK2,CCT3,CD44,EIF3G,ELAVL1,HNRNPH1,PRDX4,RPL19,RPL22,RPL27A,RPL7,RPS13,RPS19,RPS5,SNRPD2,UBE2V1,XRCC6
	PUF60	other	MYC,PUF60	2	Inhibited	-3.5	3.52E-14	0.0001	AK2,CCT3,CD44,EIF3G,ELAVL1,HNRNPH1,PRDX4,RPL19,RPL22,RPL27A,RPL7,RPS13,RPS19,RPS5,SNRPD2,XRCC6
	SGI 1776	chemical drug	CDKN1A,MYC,SGI 1776	2	Inhibited	-4	1.69E-13	0.0001	AK2,CCT3,CD44,EIF3G,ELAVL1,HNRNPH1,PRDX4,RPL19,RPL22,RPL27A,RPL7,RPS13,RPS19,RPS5,SNRPD2,XRCC6
WM5 (cyan)									
	F2R	G-protein coupled receptor	Akt,AKT1,EGFR,ERBB2,F2R,NOS3,PI3K (complex),PTPN6,RAC1,TGFB1	2	Activated	3.357	0.000692	0.0096	ABCF1,ADH5,EIF6,GSPT1,GSS,PHGDH,PIN1,PPIA,PRKAR1A,PSMB7,RAB18,RBBP7,S100A10,TPD52L1,UBE2V2
	ITGB4	transmembrane receptor	ABL1,AKT1,APP,CD28,EGFR,ERK1/2,ESR1,ITGB4,LMNA,Mapk,MKNK1,MTOR,MYC,NFE2L2,PGR,PI3K (complex),PTEN,PTK2,PTPN11,RAC1,SOX2,SRC,SREBF1,STAT3,TFEB	3	Activated	3.266	0.000113	0.0091	ABCF1,ATP5IF1,CGN,EIF3E,EIF6,GALK1,GSPT1,GSS,NSFL1C,PIN1,PPCS,PPIA,PREP,PRKAR1A,PSMB7,RAB27B,RBBP7,RPL12,SRSF10,SUN1,TNPO1,TPD52L1,VBP1,VPS26A
	FGFR2	kinase	Akt,EGFR,ERBB2,ERBB3,FGFR2,RB1	2	Activated	3.162	0.00124	0.0029	EIF6,GSS,OPA1,PHGDH,PIN1,PPIA,PRKAR1A,PSMB7,RBBP7,TOMM22
	SRPK1	kinase	Akt,ERBB2,FLT1,FOS,L-serine,MAPK14,SRPK1,TFEB,UBD,VDR	2	Activated	2.714	0.00208	0.008	EEF1A1,EIF6,GSS,PHGDH,PIN1,PRKAR1A,RBBP7,S100A10,SERPINB6,TOMM22,VPS26A
	imatinib	chemical drug	Akt,CREB1,ESR1,imatinib,KITLG,LCK,MYC,p70 S6k,RB1,STAT1,TP53	2	Inhibited	-2.837	0.0000589	0.0096	ABCF1,ADH5,ATP5IF1,CGN,EEF1A1,EIF6,GSPT1,GSS,PHGDH,PPIA,PREP,PRKAR1A,RAB18,RBBP7,RPL12,S100A10,SHTN1,SRSF10,TNPO1,TOMM22, TPD52L1
	emodin	chemical drug	EGFR,emodin,ERBB2,NOS2,NOS3,PTEN	2	Inhibited	-2.887	0.00185	0.0086	ADH5,EIF6,GALK1,GSPT1,GSS,PHGDH,PIN1,PPIA,PRKAR1A,PSMB7,RBBP7,SRSF10
	lovastatin	chemical drug	Akt,AR,EGFR,HRAS,lovastatin,MYC,NOS2,RB1,STAT4,STAT6,TP53	2	Inhibited	-2.982	0.000105	0.0081	ABCF1,ADH5,ATP5IF1,EEF1A1,EIF6,GSS,OPA1,PHGDH,PIN1,PPIA,PREP,PRKAR1A,PSMB7,RBBP7,RPL12,S100A10,SUN1,TOMM22,TPD52L1
	AEE 788	chemical drug	AEE 788,Akt,EGFR,ERBB2, ERBB3,FLT1	2	Inhibited	-3	0.00179	0.0033	EIF6,GSS,PHGDH,PIN1,PPIA,PRKAR1A,PSMB7,RBBP7,TOMM22
	sitravatinib	chemical drug	AKT1,ANXA2,ANXA7,ATF4,AXL,DDR2,EGFR,ELK1,ERBB3,ESR1,FLT1,FLT4,HRAS,JUN,KDR,KIT,KRAS,LCK,MET,MTOR,NGF,p70 S6k,Pdgfr,PI3K (complex),PTPN6,RAC1,RET,sitravatinib,STAT1,Trk Receptor	3	Inhibited	-3.441	0.0000801	0.0084	CGN,EEF1A1,EIF6,GSPT1,OPA1,PHGDH,PIN1,PPIA,PRKAR1A,PSMB7,RBBP7,RPL12,S100A10,SERPINB6,SHTN1,TNPO1,TOMM22,TPD52L1,UBE2V2
	3D185	chemical drug	3D185,Akt,CSF1R,EGFR,EIF4E,ERBB2,ERBB3,FGFR1,FGFR2,FGFR3,MYC,NFE2L2,PI3K (complex),PTPN6,RB1	3	Inhibited	-4.243	0.000163	0.0099	ABCF1,ATP5IF1,EEF1A1,EIF3E,EIF6,GSPT1,GSS,OPA1,PHGDH,PIN1,PPIA,PREP,PRKAR1A,PSMB7,RBBP7,RPL12,TOMM22,UBE2V2
WM6 (tan)									
	EDN1	cytokine	Akt,AKT1,AMPK,Ap1,arachidonic acid,c-Src,CaMKII,CDK1,CDK2,Creb,CREB1,CSF2,CXCL12,E2F3,EDN1,EGFR,ERBB2,ERK,ERK1/2,ESR1,EZH2,FOXO3,GLI1,H/K/NRAS,HBEGF,HDAC4,Hif1,HRAS,IFNG,IGF1R,IL13,INHBA,IRF4,Jnk,JUN,Mapk,MAPK1,MAPK14,MAPK3,MAPK8,Mek,mir-21,MMP1,MMP13,MMP14,MMP3,MMP9,MTORC1,MYC,NEDD9,NFkB (complex),NRAS,P38 MAPK,Pka,Pkc(s),PRKCA,PRKD1,PTK2,RAC1,RAF1,RAS,RGS2,Rock,RPS6KA3,SMARCA4,SP1,SPP1,SRC,STAT3,TCF7L2,TP63,XBP1	3	Activated	3.182	0.0000248	0.0046	AHCY,ATP5MG,BAG3,CELF1,CHMP4B,CRIP2,CRYZ,CSDE1,DTX3L,ENSA,GALNT2,GCDH,GOLPH3L,HNRNPM,HTRA1,IGF2R,LDHA,LPCAT1,MAL2,PCYT1A,PDHB,PHB,PITRM1,PYCR1,RBBP9,RPL28,SLC3A2,STRAP,TWF1,TXNRD1,WFS1,YKT6
	NOX4	enzyme	Akt,AKT1,INSR,Jnk,NFE2L2,NOX4,STAT3	2	Activated	3.051	0.000402	0.0061	ATP5MG,CLPTM1L,COPS5,CPT1A,CRIP2,CRYZ,HNRNPM,IGF2R,LDHA,PDHB,PHB,SLC3A2,TXNRD1
	Pri3d1 (includes others)	other	AKT1,Creb,CREB1,EGFR,EIF2AK3,ESR1,FLI1,Jnk,JUN,mir-21,MYC,NFE2L2,NFKB1,PI3K (complex),PRKCD,Pri3d1 (includes others),STAT3,TP53,TP63	3	Activated	2.887	0.0000087	0.0041	BAG3,CELF1,CLPTM1L,COPS5,CRIP2,CRYZ,CSDE1,DNM1,ENSA,GNAI3,HNRNPM,HTRA1,IGF2R,LDHA,PDHB,PHB,PRDX2,PYCR1,RPL28,SLC3A2,SNU13,STRAP,TIMM44,TXNRD1,VPS35,WFS1,YKT6
	taurocholic acid	chemical - endogenous mammalian	Akt,AKT1,AMPK,EGFR,Jnk,PI3K (complex),STK11,taurocholic acid	2	Activated	2.887	0.000703	0.0092	AHCY,CELF1,CPT1A,CRIP2,CRYZ,HNRNPM,PDHB,PRDX2,PYCR1,PYCR2,SLC3A2,TXNRD1
	AATF	transcription regulator	AATF,AKT1,E2F1	2	Activated	2.828	0.0000311	0.0003	CRIP2,CRYZ,CSDE1,HNRNPM,PHB,RAB1A,SERPINF2,SLC3A2
	D-thioctic acid	chemical - endogenous mammalian	AKT1,D-thioctic acid,INSR,PI3K (complex)	2	Activated	2.828	0.0000796	0.0001	ATP5MG,CPT1A,CRYZ,HNRNPM,IGF2R,PDHB,PRDX2,SLC3A2
	Vegf	group	Akt,AKT1,AMPK,BAD,BECN1,CSF2,CXCL12,EGFR,ERBB2,ERK,ERK1/2,ERN1,ESR1,FLT1,FOXO3,HRAS,IGF1R,IKZF1,IL13,IL1B,INSR,IRF4,Jnk,KRAS,MAP2K1,MAP2K3,Mapk,MAPK1,MAPK14,MAPK3,MAPK7,Mek,mir-21,MMP9,MYC,NEDD9,NFE2L2,NFkB (complex),NR4A1,NRAS,P38 MAPK,PAK1,PAK2,PI3K (complex),PITX2,PKD1,PRKCG,PRKD1,RAC1,RAF1,RAS,RET,RGS2,SPP1,SRC,STAT3,SYK,TCF7L2,TP63,Vegf,XBP1	3	Activated	2.475	0.0000172	0.0045	AHCY,ATP5MG,CELF1,CHMP4B,CLPTM1L,COPS5,CPT1A,CRIP2,CRYZ,CSDE1,DTX3L,ENSA,GALNT2,GCDH,GOLPH3L,HNRNPM,HTRA1,LDHA,LPCAT1,MAL2,PCYT1A,PDHB,PHB,PITRM1,RBBP9,SERPINF2,SLC39A11,SLC3A2,STRAP,TWF1,VPS35,YKT6
	SD-1008	chemical - kinase inhibitor	AKT1,AMPK,CDKN1B,Creb,EGFR,ERBB2,ESR1,FHIT,HRAS,IGF1R,IRF4,JAK2,Jnk,KRAS,mir-21,MKNK1,MUC1,MYC,NEDD9, NFKB1,PKD1,SD-1008,SP1,SRC,STAT3, TP63	3	Inhibited	-2.502	0.0000136	0.0054	AHCY,ATP5MG,BAG3,CELF1,COPS5,CRIP2,CRYZ,CSDE1,ENSA,GALNT2,GNAI3,HNRNPM,LDHA,LPCAT1,MAL2,PCYT1A,PDHB,PHB,PYCR1,RAB1A,RPL28,SERPINF2,STRAP,TXNRD1,VPS35,WFS1,YKT6
	mir-149	microRNA	AKT1,E2F1,mir-149	2	Inhibited	-2.828	0.0000421	0.0002	CRIP2,CRYZ,CSDE1,HNRNPM,PHB,RAB1A,SERPINF2,SLC3A2
	FMR1	translation regulator	AKT1,EIF4E,FMR1,MTOR,PI3K (complex)	2	Inhibited	-3	0.000631	0.0026	ATP5MG,BAG3,CRYZ,DNM1,HNRNPM,LDHA,PHB,PRDX2,SLC3A2
WM11 (red)									
	SLC2A4	transporter	INSR,IRS1,Irs3,MAPK1,SLC2A4	2	Activated	3.742	0.00000215	0.0023	ACADS,ATP5PD,CRAT,DDX58,ECI1,FASN,HLA-B,ISG15,LGALS3BP,NDUFA9,NDUFB4,OAS2,STAT1,TAP1
	Type I BMP receptor	group	Alp,BMP7,BMPR1B,CBL,Creb,P38 MAPK,PPARG,PRL,SMAD1,SMAD1/5/9,TP53,Type I BMP receptor	3	Activated	3.402	9.07E-08	0.0041	ACAA1,ACADS,ATP5PD,CDK1,COX5A,CRAT,DDX58,EMILIN1,FASN,FKBP3,FKBP4,FLNB,GLUL,GNAI1,HLA-B,HMGN2,HSPB1,ILK,KPNB1,KRT18,NDUFS1,NPEPPS,OAS2,PCK2,PDRG1,PSAP,QDPR,STAT1
	IL2RG	transmembrane receptor	AR,BCAR1,CD28,CTNNB1,ESR1,estrogen receptor,FLT1,FOS,IFNG,IGF1R,IL2RG,IL4,INSR,IRF3,JAK1,JAK3,MTOR,MYC,PTK,PTPN11,RET,Sfk,SIRT1,STAT1,STAT3,STAT4,STAT5A,STAT6,TP53,VAV1,VAV2	3	Activated	3.13	4.43E-08	0.0072	ACAA1,ATP6V0D1,BANF1,CDK1,COX5A,CRAT,DDB1,DYNC1I2,ECI1,EMILIN1,EPPK1,ERMP1,FASN,FKBP3,FKBP4,GDI2,GSTM3,H1-0,HMGN2,IGHM,ILK,KDM4B,LAMTOR3,LGALS3BP,MARCKS,NDUFB4,NDUFS1,NPEPPS,NUCB1,OAS2,PCMT1,PDRG1,PIP,PSAP,PTMS,QDPR,RAB11B,RALA,SLC9A3R1,STARD10,SUMO2,SYNGR2,TAP1,TSTA3,WARS1
	ACVRL1	kinase	ACVRL1,Alp,AR,Creb,CXCL12,MAPK1,MMP14,MTOR,PPARG,PRL,SKIL,SMAD1/5/9,SMAD2,SRC,STAT3,TGFB1	3	Activated	2.828	6.83E-08	0.0093	ACAA1,ACADS,ARPC4,ATP5PD,BCAM,CDK1,COX5A,CRAT,DDB1,DDX58,EMILIN1,EPPK1,FBN1,FKBP4,FLNB,HMGN2,HNRNPA1,HSPB1,IGHM,ILK,LGALS3BP,MARCKS,NPEPPS,PCDH11X,PCK2,PCMT1,PIP,RAB11B,SRSF6,STARD10,STAT1,WARS1
	SCLT1	transporter	BCAR1,MAPK1,SCLT1	2	Activated	2.828	0.000115	0.0071	DDX58,HLA-B,ISG15,LGALS3BP,OAS2,RALA,STAT1,TAP1
	INSR	kinase	INSR	1	Activated	2.646	0.0000215	0.0013	ACADS,ATP5PD,CRAT,ECI1,FASN,NDUFA9,NDUFB4
	IP6K2	kinase	AKT1,IP6K2,MAP3K7,STK11,TP53	2	Inhibited	-2.746	1.73E-07	0.0053	ACADS,CDK1,COX5A,DDX58,EMILIN1,FASN,FKBP3,FKBP4,GLUL,GNAI1,HLA-B,HMGN2,HSPB1,ILK,KPNB1,KRT18,NDUFA9,NDUFS1,NPEPPS,OAS2,PCK2,PDRG1,PSAP,QDPR,STARD10,TAP1
	GDF7	growth factor	Alp,CBL,Creb,GDF7,P38 MAPK,PRL,SMAD1,TP53	3	Inhibited	-2.746	3.47E-07	0.008	ACADS,ATP5PD,CDK1,COX5A,DDX58,EMILIN1,FASN,FKBP3,FKBP4,FLNB,GLUL,GNAI1,HLA-B,HMGN2,HSPB1,ILK,KPNB1,KRT18,NDUFS1,NPEPPS,OAS2,PDRG1,PSAP,QDPR,STAT1,TAP1
	YWHAG	other	FOXO1,MAPK1,MAPK14,TP53,YWHAG	2	Inhibited	-2.887	2.43E-07	0.0083	ACADS,CDK1,COX5A,DDX58,EMILIN1,FASN,FKBP3,FKBP4,FLNB,GLUL,GNAI1,HLA-B,HMGN2,HSPB1,ILK,KPNB1,KRT18,LGALS3BP,NDUFA9,NDUFS1, NPEPPS,OAS2,PCK2,PDRG1,PSAP,QDPR,STAT1
	MTORC1	complex	Creb,GSK3B,MTOR,MTORC1,RELA,RICTOR,SREBF1,TFEB,TP53	2	Inhibited	-2.921	1.88E-07	0.0096	ACADS,ATP5PD,ATP6V0D1,ATP6V1A,CDK1,COX5A,EMILIN1,FASN,FKBP3,FKBP4,FLNB,GLUL,GNAI1,HLA-B,HMGN2,HSPB1,IGHM,ILK,KPNB1,KRT18, MAPRE1,NDUFA9,NDUFB4,NDUFS1,NPEPPS,PDRG1,PSMA3,QDPR,RAB11B,TAP1
WM20 (darkgrey)									
	PAK2	kinase	ERK,MAPK9,MYC,PAK2,RAF1	2	Activated	2.333	4.82E-07	0.0054	ALB,HSP90AA1,HSPA9,LMNA,RAN,RPL6,RPS16,RPS7,TUBA1A
	NFE2L2	transcription regulator	NFE2L2	1	Activated	2.236	0.00000829	0.0013	HSP90AA1,HSPA9,LMNA,RAN,RPS16
	HMGCR	enzyme	HMGCR,MYC,YAP1	2	Activated	2.121	8.91E-07	0.0025	ALB,HSP90AA1,HSPA9,LMNA,RPL6,RPS16,RPS7,TUBB
	Max-Myc	complex	CDKN1B,Max-Myc,MYC	2		1.89	0.00000459	0.0029	ALB,CCT6A,HSP90AA1,HSPA9,RPL6,RPS16,RPS7
	BRD4	kinase	BRD4,MYC	2		1.89	0.00000525	0.0032	ALB,HSP90AA1,HSPA9,RPL6,RPS16,RPS7,TUBB
	MLXIPL	transcription regulator	MLXIPL	1		1.732	0.0000707	0.0002	RPL6,RPS16,RPS7
	CCNE1	transcription regulator	CCNE1,CDKN1B,ID2,MYC,POU5F1,SKP2,SOX2	2		1.667	0.0000018	0.0037	ALB,CCT6A,HSP90AA1,HSPA9,PHB2,RPL6,RPS16,RPS7,TUBA1A
	Insulin	group	Insulin	1		1.633	1.54E-07	0.0001	ALB,CCT6A,HSP90AA1,HSPA9,RPS16,TCP1
	INSIG2	other	ERK,HMGCR,INSIG2,MYC,SREBF2,YAP1	3		-1.667	0.00000021	0.0021	ALB,HSP90AA1,HSPA9,LMNA,RPL6,RPS16,RPS7,TUBA1A,TUBB
	UBA2	enzyme	UBA2,Ubiquitin	2		-1.732	0.0000726	0.0003	ALB,HSP90AA1,HSPA9
	CTDSP1	phosphatase	CTDSP1,MYC,TWIST1	2		-1.89	0.00000504	0.0034	ALB,HSP90AA1,HSPA9,LMNA,RPL6,RPS16,RPS7
	FBXL14	enzyme	FBXL14,MYC,TWIST1	2		-1.89	0.00000648	0.0041	ALB,HSP90AA1,HSPA9,LMNA,RPL6,RPS16,RPS7
	PFDN5	transcription regulator	MYC,PFDN5,TP73	2		-1.89	0.0000149	0.0081	ALB,HSP90AA1,HSPA9,RAN,RPL6,RPS16,RPS7

Table S2. The top 10 master regulators and participating regulators of the COLD-characteristic network modules (WM7, WM12, WM18, and WM24), which were predicted to have high values of activation/inhibition |z-score| using the Ingenuity Pathway Analysis (IPA). Chemical drugs are indicated in red letter.

Modue number (color)	Master Regulator	Molecule Type	Participating regulators	Depth	Predicted Activation	Activation z-score	p-value of overlap	Network bias-corrected p-value	Target molecules in dataset
WM7 (Green)									
	trichosanthin	chemical drug	AKT1,BAX,CCR1,CCR2,CCR5,CDKN1A, CEBPA,CXCR4,EIF4E,ERK,G protein,IL6, MAPK3,NFKBIA,NPAT,P38MAPK,PGR, PRKAA1,PTEN,RELA,SMAD3,TGFB1,TP53, TP73,trichosanthin,TSC2, ZAP70	3	Activated	3.266	5.45E-10	0.0137	ALDH1A1,ANXA1,ANXA6,ARHGDIB,ARPC1B,ARPC2,ATP6V1B2,CAP1, CAT,CFL1,COL6A1,COL6A2,COL6A3,CORO1A,CORO1C, COTL1,CSK, CTSS, DYNLL1,GDI1,GMFB,GNL3 ,GSR,GSTO1,H1-10,H1-5,HBD,HCLS1,HLA-DQB1, IFI16,IL16,KRT15,LCP1, LGALS9B,LSP1, MATR3,MVP,PDHA1,PFN1,PTPN6,RAB8A,RAC2,RHOA,RHOG,SERPINB9,SLC4A1,THY1,TRA2A,UBE2L3,VASP
	chrysotile asbestos	chemical toxicant	AKT1,APP,CDKN1A,CEBPA,CEBPB,chrysotile asbestos,CXCL12,EIF4E,ERK, ESR1,FOS,HNF4A,JUN/JUNB/JUND, MKNK1,MMP2,MMP9,MYC,MYCN,NFKBIA, NPAT, P38 MAPK,PGR,SMAD3,SRF, STAT6,STIM1, TGFB1, TNF,TNFRSF1A, TP53,TP73,TSC2	3	Activated	3.138	8.61E-11	0.0026	ALDH1A1,ANXA1,ANXA11,ANXA6,ARPC1B,ARPC2,ATP6V1B2,CAP1,CAT,CFL1,COL6A2,CORO1A,CORO1C,CSK,CTSS,CYFIP1, DYNLL1, EFHD2,GDI1,GMFB,GNL3,GPX4,GSR,GSTO1,H1-10,H1-5,H3-3A/H3-3B,H4C1,HBD,HCLS1,HMGA1,IFI16,IL16,ITGB2, KCTD12,KRT15,LCP1, LGALS9B,LSP1,MATR3,MVP,PDHA1,PFN1,PNP,PSMA2,PTBP1,PTPN6,RAB8A,RAC2,RHOA,RHOG, RPL9,SEC11A,SERPINB9,SLC4A1,THY1, UBE2L3,VASP,WDR1
	TSC22D1	transcription regulator	AKT1,AR,BAX,BCL2L1,CCND1,CDKN1A, CEBPA,EIF4E,FOXO1,FOXO3,IL6,MAPK3, NFKBIA,NPAT,P38MAPK,PGR,PPARGC1A,PRKAA1,RELA,SMAD2,SMAD3,STK11, TGFB1,TGM2,TP53,TP73,TSC2,TSC22D1	3	Activated	2.746	1.2E-11	0.0036	ALDH1A1,ANXA1,ANXA6,ARHGDIB,ARPC1B,ARPC2,ATP6V1B2, CAP1,CAT,CFL1,COL6A1,COL6A2,COL6A3,CORO1A,CORO1C, COTL1,CSK,CTSS,DYNLL1,EHD1,GCA,GDI1,GMFB,GNL3,GSR, GSTO1,H1-10,H1-5,HBD,HLA-DQB1,IFI16,IL16,KRT15,LCP1, LGALS9B,LSP1, MATR3,MVP,NDUFS8,PDHA1,PFN1,PTPN6, PTPRC,RAB8A,RAC2,RAP1A,RHOA,RHOG,SERPINB9,SLC4A1, THY1,UBE2I, UBE2L3,VASP
	CDK9	kinase	AKT1,AR,ATF4,BRD4,CDK9,CDKN1A, CEBPA,CEBPB,EIF4E,EP300,EPO,FOXA2,GATA4,MAPK1,MBP,MYC,MYOD1,NFE2L2,NFK BIA,NPAT,NR4A1,PGR,PRKAA1,PTEN,RB1,RCHY1,RELA,SMAD3,TP53,TP73, TSC2	3	Activated	2.711	1.55E-11	0.0027	ANXA1,ANXA6,ARHGDIB,ARPC1B,ATP6V1B2,CA1,CAP1,CAT, COL6A2,CORO1A,CSK,DYNLL1,EFHD2,EHD1,GDI1,GMFB, GMFG,GNL3, GSR,H1-10,H1-5,H3-3A/H3-3B,H4C1,HBB,HBD,HCLS1, HDAC1,IFI16,IL16,ITGB2,KRT15,LCP1,LGALS9B,LMNB2, LSP1,MATR3,MVP,NDUFS8, PDHA1,PFN1,PNP,PTBP1, PTPN6,PTPRC, RAB8A,RAC2,RAP1A,RHOA, RHOG,RPL9,SERPINB9, SLC4A1, THY1,UBE2L3
	4-hydroxynonenal	chemical toxicant	4-hydroxynonenal,Akt,AKT1,CDKN1A, CXCL8,ERN1,F3,IL6,MAPK1,NFE2L2, NFKBIA,P38 MAPK,PI3K (complex),PPARG, PTGS2,SIRT1, STK11,TGFB1,TNF	2	Activated	2.132	2.91E-11	0.0078	ANXA1,ANXA11,ARHGDIB,ARPC2,CFL1,COL6A1,COL6A2,COL6A3,CORO1A,CORO1C,COTL1,CSK,CTSS,DYNLL1,EFHD2, EHD1,GDI1,GMFB,GPX4,HBA1/HBA2,HBB,HDAC1,ITGB2,KRT15,LCP1,LGALS9B,LTA4H,MATR3,NDUFS8,PDHA1,PEA15,PNP, PSMA2,PTPN6,PTPRC,RAP1A, RHOA,SEC11A,SERPINB9,SLC4A1,SNRNP70,THY1,UBE2I,UBE2L3
	RBBP4	enzyme	AKT1,CDKN1A,CEBPA,CEBPB,E2F1, EIF4E,ERK,FOS,JUN/JUNB/JUND,KRAS, MAP3K8,MAPK1,MKNK1,MYC,MYCN,NPAT,NR4A1,NRAS,PGR,PTGS2,RAS,RASSF5, RBBP4,SMAD3,SPARC,SRF,STAT6,STIM1,TNFRSF1A,TNFSF11,TP53,TP73	3		1.961	3.61E-11	0.001	ALDH1A1,ANXA1,ANXA6,ARPC1B,ARPC2,CAP1,CAT,COL6A2,CORO1A,CTBP1,DYNLL1,EFHD2,EHD1,GCA,GMFB,GNL3,GSR, GSTO1,GZMK,H1-10,H1-5,H3-3A/H3-3B,H4C1,HBD,HMGA1,IFI16, IL16,ITGB2,KRT15,LCP1,LGALS9B,LSP1,LTA4H,MATR3, MVP,NDUFS8,PDHA1,PFN1, PPP1R7,PSMA2,PTBP1,PTPN6,RAB8A,RAC2,RHOA,RHOG,RPL9, SEC11A,SERPINB9,THY1, U2AF1/U2AF1L5,UBE2I,UBE2L3,VASP,WDR1
	fluticasone propionate	chemical drug	AKT1,AR,CEBPB,ERK,fluticasone propionate,FOS,GATA1,IFNG,Ige,IL4,IL5, IL6,INSR,JAK2,LIF,MAPK1,MAPK8,NR3C1,P38MAPK,REL,RELA,SFPQ,SMAD3,SOX4,STAT 4,TP53,TP73	3	Inhibited	-2.2	1.18E-11	0.0031	ALDH1A1,ANXA1,ANXA6,ARHGDIB,ARPC1B,ARPC2,CA1,CAP1,CAT,COL6A3,COTL1,CYFIP1,EFHD2,EHD1,FGL2,GDI1,GNL3, GPX4,H1-10,H2AC7,H2BC3,H3-3A/H3-3B,H4C1,HMGA1,IDH3A,IFI16,IL16,ITGB2,KCTD12,LCP1,LSP1,LTA4H,MATR3,MVP, PEA15,PFN1,PNP, PSMA2,PTPRC,RAB8A,RAC2,RHOA,RPL9,SEC11A,SERPINB9,SF3B2,SLC4A1,SNX1,THY1,TRA2A, U2AF1/U2AF1L5,UBE2I,UBE2L3,VASP
	ANGPTL4	other	Akt,AKT1,ANGPTL4,AR,E2F1,FOS,GATA1,GC-GCR dimer,HIF1A,JAK2, JUN,JUN/JUNB/JUND, LIPE,LPL,P38 MAPK,RELA,SP1,SPI1,STAT1,TGFB1,TP53	3	Inhibited	-2.294	1.45E-11	0.0059	ALDH1A1,ANXA1,ANXA6,ARHGDIB,ARPC1B,ARPC2,CA1,CFL1,COL6A2,COL6A3,CORO1A,CORO1C,COTL1,CYFIP1,GDI1, GNL3,HBA1/HBA2, HDAC1,HLA-DQB1,HMGA1,IDH3A,IFI16,KCTD12,KRT15,LGALS9B,LSP1,MATR3,MSN,MVP,PDHA1,PEA15, PFN1,PSMA2,PTPN6,PTPRC, RAB8A,RAC2,RAP1A,RHOA,RPL9,SERPINB9,SLC4A1,SNRNP70,THY1, UBE2L3,VASP
	JUN	transcription regulator	Akt,AR,E2F1,FOS,GATA1,GC-GCR dimer, HIF1A,JUN,JUN/JUNB/JUND,MAPK9, P38MAPK,SP1,SPI1,TGFB1,TP53	2	Inhibited	-2.4	5.36E-11	0.0091	ANXA1,ANXA6,ARPC1B,ARPC2,CA1,CFL1,COL6A2, COL6A3,CORO1A, CORO1C,COTL1,CYFIP1,FGL2,GDI1,GNL3, HBA1/HBA2,HDAC1,HLA-DQB1,HMGA1,IDH3A,IFI16,IL16,ITGB2,KRT15,LGALS9B,LSP1,MSN,MVP,PDHA1,PEA15,PFN1, PSMA2,PTPN6,PTPRC,RAB8A,RAC2,RAP1A, RHOA,RPL9,SERPINB9,SLC4A1,SNRNP70,THY1,VASP
	SGI 1776	chemical drug	AKT1,BAD,BCL2L1,CDKN1A,CSF2,EIF4E,EIF4EBP1,EP300,F2,FOS,GLI1,HSF1,MAP3K8,M YC,NFE2L2,NFKBIA,NPAT,PIM1,PIM2,PTEN,RAF1,RUNX1,SGI 1776,STAT6,Tgf beta,TP53,TP73,TSC2	3	Inhibited	-2.449	5.61E-11	0.0038	ANXA1,ANXA6,ARPC1B,ATP6V1B2,CAP1,COL6A1,COL6A2,COL6A3,CORO1A,CORO1C,COTL1,CSK,DYNLL1,EFHD2,GCA, GMFB,GMFG,GNL3,GSR,GSTO1,H1-5,H3-3A/H3-3B,HBA1/HBA2,HBB,HCLS1,HLA-DQB1,HMGA1,IL16,KCTD12,LCP1,LSP1, MATR3,MVP,PDHA1,PFN1,PNP,PSMA2,PTBP1,PTPRC,RAB8A,RAC2,RAP1A,RHOA,RHOG,RPL9,SERPINB9,THY1,UBE2I, UBE2L3
WM18 (Black)									
	MAPK3	kinase	Akt,APP,ARRB1,ATF2,CCND1,CREM,CSF1,DUSP1,ERK1/2,FOS,HIF1A,Mapk,MAPK1,MAP K3,MKNK1,NFE2L2,NFkB (complex), NFKBIA,PARP1,STAT1,STAT3,TFCP2, TP53,TWIST1,XBP1	2	Activated	3.536	4.07E-09	0.0338	ACTB,ACTN4,AGT,AHSG,ANXA4,APCS,C4A/C4B,CFB,CFH,CKB,CP,CRYAB,DNAJC3,ENAH,GART,GLS,GSTK1,KNG1,MFGE8, NAD+,NIBAN2,PDIA4,PDIA6,PDXK,PFKL,PSMD5,RUVBL1,S100B,TAPBP,TBCA, TM9SF2,TUBB3
	APP	other	ACE,AMPK,APP,C3,CFB,Creb,ERK1/2,FOS,FOXO1,FOXO3,HIF1A,INSR,MAPK1,MTOR,NFk B (complex),NFKBIA,NOTCH1,P38 MAPK,PARP1,Pkc(s),RAC1,STAT3,TFCP2,TP53,TP73	2	Activated	2.611	1.13E-09	0.0087	ACTB,ACTN4,AGT,AHSG,ANXA4,APCS,APOE,ASS1,C4A/C4B,CFH,CKB,CP,DECR1,DNAJC3,ELOB,ERP29,GAA,GART,GLS, GSTK1,KNG1,MFGE8,NAD+,ORM1,PDIA6,PFKL,PYGB,S100B,SERPING1,TAPBP,TBCA, TUBB3,UGGT1
	sumatriptan	chemical drug	Akt,AR,Creb,cyclic AMP,ERK1/2,FOS, HTR1A,NFE2L2,NFkB (complex),NQO1, SP1,SPI1,sumatriptan	3	Activated	2.353	6.45E-11	0.0045	A2M,ACSL1,ACTB,AGT,ANXA4,APCS,APOE,ASS1,CALR,CFB,COX4I1,CRYAB,DNAJC3,DSG2,ERP29,GSTK1,HSP90AB1,NAD+, ORM1,PDIA4, PDIA6,PSMD5,RUVBL1,TAP2,TAPBP,TBCA
	nordihydro-guaiaretic acid	chemical drug	Akt,ERBB2,ERK1/2,MAPK1,NFE2L2, nordihydroguaiaretic acid,NPM1-ALK,P38MAPK,PPARG,STAT3	2	Activated	2.294	2.62E-09	0.0192	ACLY,ACSL1,ACTB,AHSG,APCS,APOE,ASS1,CALR,CFB,COX4I1,DNAJC3,ERP29,GART,GLS,GSTK1,HSP90AB1,NAD+,PDIA4, PDIA6, PSMD5,RUVBL1,TAP2,TAPBP
	PMEPA1	other	EGFR,MAF,NFE2L2,PMEPA1,PPARA,R-Smad,Smad,Smad2/3,SMAD3	3	Activated	2.236	3.09E-09	0.0043	ACSL1,ACTB,AGT,APCS,APOE,ASS1,COX4I1,CRYAB,DNAJC3,DSG2,ERP29,GLS,GSTK1,HSP90AB1,PDIA4,PDIA6,PSMD5, RUVBL1,S100B, SERBP1
	butylated hydroxyanisol	chemical toxicant	butylated hydroxyanisol,ERK1/2,INSR, Jnk,MAPK1,MAPK8,NFE2L2,NFKBIA, NQO1,P38 MAPK	2	Activated	2.132	5.49E-11	0.0009	A2M,ACSL1,AGT,APCS,APOE,ASS1,CALR,CFB,CP,CRYAB,DECR1,DNAJC3,GLS,HSP90AB1,MFGE8,NAD+,PDIA4,PDIA6, PSMD5,RUVBL1, TAP2,TAPBP
	MAP2K4	kinase	Akt,ATF2,ESR1,IRF7,MAP2K4,MAPK11, MAPK14,MYC,NFE2L2,NFkB (complex), NFKBIA,STAT3	2	Activated	2.043	2.94E-09	0.0261	ACTB,ACTN4,AGT,AHSG,ANXA4,APCS,CALR,CFB,CPNE1,CRYAB,DNAJC3,DSG2,ENAH,ERP29,GART,GLS,GSTK1,HPX, HSP90AB1,KIF5B,MFGE8,PDIA4,PDIA6,PFKL,PSMD5,RUVBL1,S100B,TAP2,TAPBP
	taurine	chemical - endogenous mammalian	Akt,BAX,ERK1/2,gentamicin,Mapk,MAPK8,P38 MAPK,taurine	2	Inhibited	-2.183	3.05E-09	0.0018	A2M,AGT,APCS,APOE,ASS1,C4A/C4B,COX4I1,CPQ,DECR1,GSTK1, PDIA4,PDIA6,PFDN2,RUVBL1,TAP2,TAPBP,TUBB3
	palmitoylethanol amide	chemical drug	ERK1/2,IL4,IL6,MAPK1,palmitoylethanolamide,PPARA	2	Inhibited	-2.449	1.09E-10	0.003	A2M,ACTB,AGT,ANXA4,APCS,APOE,ASS1,CALR,CFB,CFH,CP,CRYAB,DECR1,DYNC1H1,ERP29,GLS,GSTK1,HPX,IGHG3, ORM1,PFKL,TAP2,TAPBP,UGGT1
	FBXL2	enzyme	ACE,AMPK,APP,C3,CCND1,CCND3, CEBPA,CFB,Creb,ERK1/2,FBXL2,FOS, FOXO1,FOXO3,HIF1A,INSR,MAPK1,MTOR,NFkB (complex),NFKBIA,NOTCH1,P38 MAPK,PARP1,Pkc(s),RAC1,STAT3,TFCP2,TP53,TP73	3	Inhibited	-2.744	1.08E-09	0.0056	ACTB,ACTN4,AGT,AHSG,ANXA4,APCS,APOE,ASS1,C4A/C4B,CFH,CKB,CP,DECR1,DNAJC3,ELOB,ERP29,GAA,GART,GLS, GSTK1,KNG1,MFGE8,NAD+,NIBAN2,ORM1,PDIA6,PFKL,PYGB,S100B,SERPING1,TAPBP, TBCA,TUBB3,UGGT1
WM24 (Brown)									
	IGF receptor	group	26s Proteasome,AKT1,AR,CCND1,CD247, CD3,CEBPB,Creb,CREB1,EGFR,EIF4E,ERBB2,ERK1/2,ESR1,GSK3B,HIF1A,HRAS, IGF receptor,IGF1R,IGF2R,Insulin, IRS2,JAK1/2,Jnk,KRAS,MAP3K14,MAPK7,Mek,MTOR,MYC,Nfat (family),NFE2L2,NFkB (complex),NFKBIA,NOS2,NR4A1,NRAS, PI3K (complex),RAF1,RAS,SMAD3, SRF,STAT5A,STAT5a/b,TGFB1,TP53, TSC2,ZAP70	3	Activated	4.644	1.42E-17	0.0001	ABCE1,ACAA2,ACSL3,AK3,ALDH18A1,ALDH7A1,API5,ASAH1,BCAP31,C4BPA,CALU,CCT8,CDK4,CDKN2A,COL18A1,COMT, DDX3X,DEGS1,ECH1,EEF1B2,EHD4,EMD,FBL,FKBP11,FKBP1A,FKBP2,FLAD1,GGT1,GLDC,GPD1L,HM13,JPT1,LMAN2,M6PR, MANF,MAP7,MDH2,MGST2,NDUFA4, PC,PDCD6IP,PLA2G4A,PLP2,PON2,PSMA7,PSMC2,PSMD11,PSMD2,PSMD6,PTGES3, RAD23B,RCC1,REST,RHEB,RPL17,RPL30,RPL38, RPS17,RPS21,RPS26,RPS28,RPS3,RPS8,RTCB,SAR1A,SCAMP3,SHMT2, SLC25A1,SLC7A5,SND1,SOAT1,SRPRB,SSBP1,SSR3,SSR4, STEAP4,TACSTD2,TMEM43,TOP2B,TPR,UGP2,UQCRC1, UQCRC2,UQCRHL,USP14,VCP
	Grb2-Shc1-Sos	complex	26s Proteasome,AKT1,AR,CCND1,CEBPB, Creb,CREB1,EIF4E,ERBB2,ERK1/2,ESR1, FOS,GAB1,GRB2,Grb2-Shc1-Sos,GSK3B, HIF1A,HRAS,Jnk,KRAS,MAP3K14,MAPK7,Mek,MTOR,MYC,Nfat (family),NFE2L2, NFkB (complex),NKX2-1,NOS2,NR4A1, NRAS,PI3K (complex),PTEN,RAF1,RAS, RB1,Shc,SHC1,SMAD3,Sos,SRF, STAT5A,STAT5a/b,TP53,TSC2	3	Activated	3.43	7.43E-18	0.0001	ABCE1,ACAA2,ACSL3,AK3,ALDH18A1,ALDH7A1,API5,ASAH1,BCAP31,CDKN2A,COL18A1,COMT,DEGS1,ECH1,EEF1B2,EHD4, EMD,FBL, FKBP1A,FLAD1,FTL,GPD1L,H6PD,HM13,JPT1,LMAN2,M6PR,MANF,MAP7,MDH2,MGST1,MGST2,MYH10, NIPSNAP1,PC,PDCD6IP,PLA2G4A, PLP2,PON2,PSMA7,PSMC2,PSMD11,PSMD2,PSMD6,PTGES3,RANGAP1,RCC1,REST, RHEB,RPL17,RPL30,RPL38,RPS17,RPS21, RPS26,RPS28,RPS3,RPS8,RTCB,SAR1A,SCAMP3,SCO1,SERPINE2,SLC25A1, SLC7A5,SND1,SOAT1,SSBP1,SSR3,SSR4,STEAP4,SURF4, TACSTD2,TMEM43,TOP2B,TPR,TUFM,UGP2,UQCRC1, UQCRHL,USP14, VCP
	Cbp/p300	group	26s Proteasome,AR,Cbp/p300,CDKN2A,CHEK1,CREB1,CREBBP,CTNNB1,E2f,EGFR,EIF4E,EP3 00,ERBB2,ESR1,ESR2,estrogen receptor,FOS,GLI1,GSK3B,IL2,MAPK9, MYC,NEUROD1,NFE2L2,NR3C2,PPARG, PPIF,progesterone,RAF1,RELA,SMAD3, STK11,TCF4,TCF7L2,TGFB1,TGM2,TP53, TSC2,XBP1	3	Activated	3.355	8.28E-22	0.0001	ACAA2,ACSL3,AK3,ALDH18A1,ARF5,BCAP31,BID,C4BPA,CALU,CARHSP1,CDK4,COL18A1,COMT,CRELD2,DARS2,DDOST, DDX3X, DEGS1,DNM1L,ECH1,EMD,ERP44,FBL,FKBP11,FKBP1A,FKBP2,FLAD1, FMR1,FTL,GGT1,GPD1L,HM13,HSPA8,IFI44, LDHB,LMAN2,LSM3,M6PR,MAP4,MAP7,MGST1,MGST2,MME,MSLN,MT-CYB,MYH10,NAMPT,NDUFA4, NIPSNAP1,OAT,PC, PDCD6IP,PLA2G4A,PLIN3,PLP2,PPP2CA,PSMA7,PSMC2,PSMD11,PSMD2,PSMD6,PTGES3,RANGAP1,RCC1,RCN3,RHEB, RPF2,RPL30,RPL38,RPS17,RPS21,RPS26,RPS28,RPS3,RPS8,RTCB, SCAMP3,SCO1,SCPEP1,SELENOM,SERPINE2,SLC7A5, SPON2,SRPRB,SSR1,SSR3,SSR4,SURF4,TACSTD2,TAGLN2,TOP2B,TPR,TRMT10C, TUBB4A,UQCRC1,UQCRFS1,USP14, VCP,VKORC1L1
	EP300	transcription regulator	AR,CREBBP,EP300,ESR1,HIF1A,HNF4A, JUN,JUNB,NEUROD1,NFE2L2,NFkB (complex),PDX1,PPARG,SP1,STAT3, T3-TR-RXR,TP53,XBP1,ZBTB7B	2	Activated	3.333	7.24E-19	0.0001	ACAA2,AK3,AKR1C1/AKR1C2,ALDH18A1,ALDH7A1,ASAH1,BCAP31,CALU,CDKN2A,CMPK2,COL18A1,COMT,DDOST,DDX3X, DEGS1,ECH1, ERP44,FKBP11,FKBP1A,FKBP2,FLAD1,FTL,GLA,GPD1L,HM13,HSPA8, IFI44,LMAN2,M6PR,MANF,MAP4,MAP7, MGST1,MGST2,MME,MYH10, NAMPT,NDUFA4,OAT,PC,PDCD6IP,PLA2G4A,POR,PPP2CA,PSMA7, PSMD11,PSMD2,RANGAP1, RCN3,RHEB,RPS26,RPS3,SAR1A,SCAMP3, SCO1,SCPEP1,SELENOM,SERPINE2,SLC25A1,SLC7A5,SRPRB,SSBP1,SSR1, SSR3,SSR4,SURF4,TACSTD2,TAGLN2,TOP2B,TPR,UCHL1, UQCRC1,UQCRC2,UQCRFS1,USP14,VCP
	FGF3	growth factor	CCND1,CEBPB,CREB1,E2f,EGFR,ERBB2, ERK1/2,ESR1,FGF3,Fgfr,FGFR1,FOS, GADD45A,GLI1,GSK3B,HIF1A,HNF4A, HRAS,HSF1,IL6,Jnk,KAT5,KRAS,MAPK1, MAPK3,MAPK7,MAPK9,Mek,MITF,MTOR, MYC,NCOA2,Nfat (family),NFE2L2,NR4A1, NRAS,PLC gamma,PPIF,RAF1,RAS, Rsk,SHC1,STAT5A,TP53,XBP1	3	Activated	3.015	2.06E-19	0.0001	ABCE1,ACAA2,ACSL3,AK1,AK3,ALDH18A1,ASAH1,BCAP31,BID,CALU,CCT8,CDKN2A,COL18A1,DDOST,DDX3X,DNM1L,ECH1, EEF1B2,EHD4, EMD,ERP44,FKBP11,FKBP1A,FKBP2,FLAD1,FTL,GPD1L,GPD2,H6PD, HM13,HSPA8,IFI44,LDHB,LMAN2, LSM3,M6PR,MANF,MAP4,MAP7,MGST2,MT-CYB,MYH10,NAMPT,NDUFA4,NIPSNAP1,OAT,PC, PDCD6IP,PLA2G4A,POR, PPP2CA,PSMA7,PSMD1,PSMD11,PSMD2, PSMD6,RCC1,RCN3,RHEB,RPL17,RPL30,RPL38,RPS17,RPS21,RPS26, RPS28, RPS3,RPS8,RTCB,SCAMP3,SCO1,SCPEP1,SELENOM, SERPINE2,SLC25A1,SND1,SPON2,SRPRB,SSR1,SSR3,SSR4,STEAP4, SURF4,TAGLN2,TMEM43,TOP2B,TPR,TUFM,UCHL1,UGP2,UQCRC1, UQCRFS1,UQCRHL,USP14,VKORC1L1
	PPM1B	phosphatase	APP,AR,BRD4,CDK2,CDK6,CDK9,CEBPB, Creb,CSF2,CTNNB1,E2f,EIF4E,EP300, ERBB2,FOS,GADD45A,GLI1,HNF4A,HSF1, HTT,IKBKB,IL1B,MAPK3,MAPK9,MYC, NFE2L2,NFkB (complex),NOS2,NR1I2,PPIF, PPM1B,STAT5A,TBK1,TP53,TSC2,TWIST1,WT1,XBP1,ZNF335	3	Inhibited	-3.28	2.02E-18	0.0001	ACAA2,ACSL3,AK1,AK3,ALDH18A1,ALDH7A1,API5,BCAP31,CALU,CCT8,CDK4,CMPK2,COL18A1,DDOST,DDX3X,DEGS1, DNM1L,ECH1,ERP44, FKBP11,FKBP1A,FKBP2,FLAD1,FMR1,GLA,GPD1L,GPD2,HM13,HSPA8, IFI44,LDHB,LMAN2,LSM3,M6PR, MAP4,MGST2,MME,MSLN,MT-CYB,MYH10,NAMPT,NCSTN,NDUFA4,NIPSNAP1,OAT,PDCD6IP,PGRMC2,PLA2G4A, PLAA, PLIN3,PLP2,POR,PPP2CA,PSMA7,PSMD1,PSMD11, PSMD2,PTGES3,RANGAP1,RBP1,RCC1,REST,RPL30,RPL38,RPS17, RPS21,RPS26,RPS28,RPS3,RPS8,SAR1A,SCO1,SCPEP1,SELENOM, SERPINE2,SLC25A1,SLC7A5,SPON2,SRPRB,SSR1, SSR3,SSR4,SURF4, TAGLN2,TOP2B,UCHL1,UGP2,UQCRC1,UQCRFS1,USP14,VKORC1L1
	TBC1D10C	other	APP,CCND1,CEBPB,Creb,CREB1,EIF4E, ERK,ERK1/2,ESR1,ETV5,FOS,FOXO4, GSK3B,HIF1A,HSF1,MAPK3,MAPK7,MTOR,MYC,MYCN,NCOA2,Nfat (family),NFE2L2,NFkB (complex),NFKBIA, NOS2,PI3K (complex),RXRA,SHC1,SIRT1, SRF,STAT5a/b,TBC1D10C,TP53,TSC2, TWIST1,XBP1	3	Inhibited	-3.363	3E-17	0.0002	ABCE1,AK3,AKR1C1/AKR1C2,ALDH18A1,ALDH7A1,API5,BCAP31,CCT8,COMT,DDOST,DEGS1,ECH1,EEF1B2,EHD4, EMD,ERP44,FBL, FKBP11,FKBP1A,FKBP2,FLAD1,FTL,GLA,GPD1L,GPD2,HM13,HSPA8, LMAN2,M6PR,MANF,MAP7,MDH2, MGST2,MME,MSLN,NDUFA4, NIPSNAP1,PDCD6IP,PGRMC2,PLA2G4A,PLIN3,PLP2,PSMA7,PSMD11, PSMD2,PTGES3, RANGAP1,RCC1,RCN3,RHEB,RPL17,RPL30,RPL38, RPS17,RPS21,RPS26,RPS28,RPS3,RPS8,SAR1A,SCAMP3,SCO1, SELENOM,SLC7A5,SND1,SOAT1,SRPRB,SSR1,SSR3,SSR4,STEAP4, SURF4,TACSTD2,TOP2B,TPR,UCHL1,UGP2,UQCRC1, UQCRFS1,USP14, VCP
	NMS-873	chemical reagent	CCND1,DUSP1,GADD45A,GNAS,HSPA8, KRAS,MAPK3,MYC,NMS-873,NUP133, PLK4,RTN4,TP53	2	Inhibited	-3.657	1.67E-19	0.0001	ABCE1,ACAA2,ACSL3,AK1,AK3,ALDH18A1,BCAP31,BID,CMPK2,COL18A1,DDX3X,DNM1L,ECH1,EEF1B2,FBL,FKBP1A,FLAD1, GGT1,GPD1L,H6PD, IFI44,JPT1,LMAN2,MANF,MGST2,MSLN,MT-CYB,MYH10,NAMPT,NDUFA4, OAT,PC,PDCD6IP,PLA2G4A, PSMA7,PSMD1,PSMD2,RBP1,RCC1,REST, RPL17,RPL30,RPL38,RPS17,RPS21,RPS26,RPS28,RPS3,RPS8,SCO1, SCPEP1,SLC7A5,SOAT1,TOP2B,TPR,TUBB4A,TUFM,UCHL1,UQCRC1, UQCRC2,UQCRFS1,USP14,VCP
	CCS1477	chemical drug	AR,CCS1477,CREBBP,EP300,ESR1,ESR2, FOXO1,GATA1,HIF1A,HNF4A,JUN,JUNB, MYC,NEUROD1,NFE2L2,NFkB (complex),NR3C2,PDX1,PPARA,PPARG, RELA,STAT3,T3-TR-RXR,TP53,XBP1, ZBTB7B	3	Inhibited	-4.964	8.19E-22	0.0001	ABCE1,ACAA2,AK3,AKR1C1/AKR1C2,ALDH18A1,ALDH7A1,ASAH1,BCAP31,CALU,CDKN2A,COL18A1,COMT,DDOST,DDX3X, DEGS1,ECH1, ERP44,FBL,FDPS,FKBP11,FKBP1A,FKBP2,FLAD1,FTL,GGT1,GLA,GPD1L,GPD2,H6PD,HM13,HSPA8, IFI44,LMAN2,M6PR,MANF,MAP7,MGST1, MGST2,MME,MSLN,MYH10,NAMPT,OAT,PBXIP1,PC,PDCD6IP,PLA2G4A,POR, PSMA7,PSMD11,PSMD2,RANGAP1,RBP1,RCC1,RCN3,RHEB,RPL17,RPL30,RPL38,RPS17,RPS21,RPS26,RPS28,RPS3,RPS8, SAR1A,SCAMP3,SCO1,SCPEP1,SELENOM,SERPINE2,SLC25A1,SLC7A5,SRPRB,SSBP1, SSR1,SSR3,SSR4,SURF4,TACSTD2, TOP2B,TPR,UCHL1,UQCRC2,USP14,VCP
	EID1	transcription regulator	AR,CREBBP,EID1,EP300,ESR1,ESR2, FOXO1,GATA1,HIF1A,HNF4A,JUN,JUNB, MYC,NEUROD1,NFE2L2,NFkB (complex), NR3C2,PDX1,PPARA,PPARG,RELA,STAT3,T3-TR-RXR,TP53,XBP1,ZBTB7B	3	Inhibited	-4.964	8.19E-22	0.0001	ABCE1,ACAA2,AK3,AKR1C1/AKR1C2,ALDH18A1,ALDH7A1,ASAH1,BCAP31,CALU,CDKN2A,COL18A1,COMT,DDOST,DDX3X, DEGS1,ECH1, ERP44,FBL,FDPS,FKBP11,FKBP1A,FKBP2,FLAD1,FTL,GGT1,GLA,GPD1L,GPD2,H6PD,HM13,HSPA8,IFI44, LMAN2,M6PR,MANF,MAP7,MGST1, MGST2,MME,MSLN,MYH10,NAMPT,OAT,PBXIP1,PC,PDCD6IP,PLA2G4A,POR,PSMA7, PSMD11,PSMD2,RANGAP1,RBP1,RCC1,RCN3,RHEB,RPL17,RPL30,RPL38,RPS17,RPS21,RPS26,RPS28,RPS3,RPS8,SAR1A, SCAMP3,SCO1,SCPEP1,SELENOM,SERPINE2,SLC25A1,SLC7A5,SRPRB,SSBP1, SSR1,SSR3,SSR4,SURF4,TACSTD2,TOP2B, TPR,UCHL1,UQCRC2,USP14,VCP
WM12 (Green-yellow)									
	SMAD5	transcription regulator	Alp,Ap1,AR,CCND1,Creb,FOS,IGF1,IL2,JUN,Nfat (family),PRL,Rb,RB1,RBL2,RUNX2, SMAD5	3	Activated	2.53	0.00000353	0.0269	ADK,ANXA2,ARF1,BGN,CNN3,COL11A1,COL5A2,CYCS,DCN,DLAT,FN1,IFIT1,IFIT3,LUM,PCBD1,PGM2L1,PPP3CA,PSMB2, TGFBI
	tert-butyl-hydroquinone	chemical reagent	AHR,JUN,MAPK1,NFE2L2,P38 MAPK, RAF1,tert-butyl-hydroquinone	2	Activated	2.236	0.00000093	0.0042	AGL,ARF1,BGN,CBR1,COL11A1,COL5A2,CYCS,DCN,FTH1,HMGB3,IFIT1, IFIT3,PMM1,PSMB2,TGFBI,UGDH
	yangonin	chemical - endogenous non-mammalian	AHR,Creb,IRF3,JUN,NFE2L2,NFKBIA,RELA,yangonin	3	Activated	2.121	0.00000593	0.0228	ADK,ARF1,CBR1,COL11A1,COL5A2,CYCS,FN1,FTH1,GATD3A/GATD3B, IFIT1,IFIT3,LUM,PMM1,PSMB2,TGFBI,UGDH
	Angiotensin II receptor type 1	group	AGTR1,Angiotensin II receptor type 1, EGFR,ERK,MKNK1,NCF1,Nfat (family), PRKCA	2	Activated	2	0.00000371	0.0022	ANXA2,BGN,COL5A2,CYCS,FN1,HMGB3,IFIT1,IFIT3,PGM2L1,POSTN, PSMB2
	CCR2	G-protein coupled receptor	CCR2	1	Activated	2	3.44E-07	0.0001	BGN,DCN,DPT,FN1,LUM,TGFBI
	MALT1	peptidase	AHR,AR,Ca2+,CTNNB1,FOS,HIF1A,HNF4A, IRF4,MALT1,MAP4K4,MTORC1,MYC,Nfat (family),NFkB (complex),NFKB2,NFKBIA, PPARG,RAF1,REL,RELA,RICTOR,SP1, STAT3,STAT4,TP53,TRAF6	3		1.886	8.13E-07	0.0141	ADK,AGL,AK4,ANXA2,ARF1,CBR1,CNN3,COL11A1,COL5A2,FAM162A,FN1,FTH1,GATD3A/GATD3B,HINT1,HMGB3,IFIT1,IFIT3, LUM,PCBD1, PDCD6,PEBP1,PGM2L1,PMM1,PPP3CA,PSMB2,RPL3,SHROOM3,SYPL1
	Atf	group	AR,Atf,ATF1,ATF2,ATF3,ATF4,ATF6,Creb, FOS,HIF1A,IRF7,JUN,MTOR,NFE2L2, RUNX2,STAT3,TP53	3		1.732	0.0000024	0.022	ADK,AK4,ANXA2,ARF1,CBR1,CNN3,COL11A1,COL5A2,CYCS,FAM162A,FN1,FTH1,IFIT1,IFIT3,LUM,PCBD1,PPP3CA,PSMB2, PSPH,RPL23, SHROOM3
	Rp-8-pCPT-cGMPS-triethylamine	chemical reagent	EGFR,ERK,ESR1,FOS,HNF4A,HSF1,L-serine, MAPK1,MYC,MYCN,NCOA1,NFE2L2, PPARG,RAF1,Rp-8-pCPT-cGMPS-triethylamine,RUNX2,SNAI1,SP1,TP53,TSC2	3	Inhibited	-2	0.0000035	0.0404	AGL,AK4,ANP32A,ANXA2,ARF1,BGN,CBR1,CDC42BPB,CNN3,COL11A1,COL5A2,DLAT,DPT,FTH1,HINT1,HMGB3,IFIT1,IFIT3, PCBD1,PEBP1,PMM1,POSTN,PPP3CA,PSMB2,PSPH,RPL10A,RPL3,SHROOM3,TGFBI,UGDH
	TLE1	transcription regulator	AR,CEBPB,CTNNB1,HIF1A,Mek,MYC, NOTCH1,PPARG,RAF1,RBL2,RUNX2, TCF4,TCF7L2,TLE1,TP53	3	Inhibited	-2.138	0.00000121	0.0363	AK4,ANXA2,BGN,CERS2,CNN3,COL11A1,DCN,DPT,FAM162A,FN1,GATD3A/GATD3B,HMGB3,IFIT1,IFIT3,LUM,PCBD1,PMM1, PPP3CA, PSMB2,RPL10A,RPL23,RPL3,SHROOM3,SYPL1,TGFBI,UGDH
	chidamide	chemical drug	chidamide,HDAC2,HDAC3,MTOR,PPARG, RUNX2,TGFB1,TLR9	3	Inhibited	-2.333	0.00000818	0.033	ADK,ANXA2,ARF1,BGN,CNN3,COL11A1,CYCS,DCN,DLAT,DPT,HINT1, IFIT1,IFIT3,LUM,PMM1,POSTN,RPL23,TGFBI
